# Cortical Microvascular Pulsatility in the Aging Mouse Brain and the Confounding Effects of Anesthesia

**DOI:** 10.1002/advs.202519324

**Published:** 2025-11-07

**Authors:** Mia Viuf Skøtt, Elizaveta Melnikova, Eugenio Gutiérrez, Vladimir Matchkov, Leif Østergaard, Dmitry D Postnov

**Affiliations:** ^1^ Center of Functionally Integrative Neuroscience Department of Clinical Medicine Aarhus University Aarhus 8000 Denmark; ^2^ Department of Biomedicine Aarhus University Aarhus 8000 Denmark

**Keywords:** aging, anesthesia, blood flow, imaging, pulsatility

## Abstract

Abnormal cerebrovascular pulsatility is associated with white‐matter injury, blood–brain‐barrier leakage, and impaired glymphatic clearance, yet its extent in the microvasculature and aging dynamics remained obscured due to experimental and technical limitations. A multi‐modal approach for quantifying flow and diameter pulsatility in small cerebral vessels is developed and applied it longitudinally in male C57BL/6JRj mice from 18 to 81 weeks, both in awake and anesthetized conditions. In the awake state, mean perfusion and pulsatility indexes varied by <10%, indicating preserved hemodynamics until late life when arterial diameter pulsatility and venular caliber rose modestly. Anaesthesia radically changes the microvascular dynamics: isoflurane produces age‐dependent hyperemia, and both isoflurane and ketamine–xylazine double flow pulsatility and reshape diameter oscillations in drug‐specific ways. To complement the longitudinal data from males, a separate cross‐sectional comparison between sexes at 50–51 weeks of age is performed, which reveal significantly lower microvascular flow pulsatility in females despite no difference in mean perfusion. The results suggest that microvascular pulsatility remains stable during healthy aging yet can shift dramatically depending on the animal's condition, even if average cerebral perfusion is unchanged.

## Graphical abstract

Multi‐modal optical imaging reveals microvascular pulsatility in the brain cortex of awake and anaesthetized mice. Awake hemodynamics in male mice are found to be remarkably stable until late life, with flow pulsatility being significantly higher than in females, despite similar average perfusion. Common anaesthetics, such as isoflurane and ketamine‐xylazine, dramatically increase flow pulsatility, revealing a significant confounding factor.

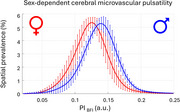

## 1 Main

Emerging evidence points to disruptions in microvascular pulsatility—the subtle rhythmic expansions and contractions of small blood vessels during the cardiac cycle— as a potentially pivotal yet understudied contributor to brain pathology and dementia development.^[^ ^1^, ^2^, ^3^, ^4^ ^]^ Increased pulsatility may mechanically damage fragile vessels, compromising the blood‐brain barrier (BBB) and promoting white matter injury.^[^ ^5^, ^6^ ^]^ Elevated pulsatility in the internal carotid or middle cerebral arteries is associated with increased incidence of white matter hyperintensities (WMH), which are predictors of cognitive decline and the development of dementia and stroke.^[^ ^6^, ^7^, ^8^ ^]^ Aging and vascular disease risk factors, such as hypertension and diabetes, are associated with loss of arterial elasticity, which may lead to more pulsatile energy dissipating in the brain microvasculature and contribute to microvascular and tissue injury.^[^ ^9^, ^10^ ^]^ These risk factors are also associated with cerebral small vessel disease (CSVD), characterized by morphological and functional changes in small cerebral arteries, arterioles, capillaries, and venules.^[^ ^11^, ^12^, ^13^, ^14^, ^15^, ^16^ ^]^ These changes include vascular stiffening and impaired blood flow responses to vasodilator stimuli and the occurrence of WMH.^[^ ^17^ ^]^ Increased microvascular pulsatility may represent a missing link between these abnormalities and a high risk of stroke and dementia^[^ ^4^, ^18^ ^]^ in CSVD patients. Reduced pulsatility, on the other hand, was suggested to disrupt essential brain clearance pathways, allowing toxic proteins to accumulate and worsen cognitive impairment in Alzheimer's disease.^[^ ^19^, ^20^, ^21^, ^22^ ^]^ Combined, these factors suggest that microvascular pulsatility has some optimal range, shifting from which may lead to cerebral dysfunction and cognitive decline via various pathways.

Characterizing the transmission of cardiac pulsation through different brain regions and its impact on brain microvasculature is pivotal for understanding brain vascular function and has broad clinical appeal. Several approaches and techniques have been used to quantify vascular pulsatility in animal models and humans. Carotid‐femoral pulse wave velocity is a gold standard measurement of arterial stiffness, which, however, only provides systemic information and no direct insight into cerebrovascular beds.^[^ ^23^ ^]^ Cerebrovascular pulsatility index is typically acquired with transcranial Doppler ultrasound^[^ ^24^, ^25^, ^26^ ^]^ and time‐of‐flight or phase contrast magnetic resonance imaging.^[^ ^27^ ^]^ While invaluable, these techniques are fundamentally limited to resolving flow and pulsatility in large cerebral arteries (typically <100–200 μ m in diameter),^[^ ^28^ ^]^ although a recent MRI‐based approach suggests a way to indirectly assess volumetric pulsatility at the microvascular level.^[^ ^29^ ^]^ Nevertheless, they lack the spatial resolution to directly probe the hemodynamics of the microcirculation ‐ the arterioles, capillaries, and venules ‐ where pulse pressure is dissipated and vital neurovascular processes occur. Recent advances in optical imaging have enabled more comprehensive methods for measuring microvascular pulsatility in animal models' brain cortex^[^ ^30^ ^]^ and human retina.^[^ ^31^, ^32^ ^]^ Technologies such as two‐photon microscopy (TPM), optical coherence tomography, laser Doppler holography, and laser speckle contrast imaging (LSCI) provide detailed, high‐resolution images of microvascular dynamics in vivo.^[^ ^30^, ^31^, ^32^, ^33^, ^34^, ^35^, ^36^ ^]^ These methods allow for the observation of pulsatility at both macroscopic and microscopic scales, providing deeper insights into the regional effects of pulsatility changes in the brain vasculature. Nevertheless, simultaneous full‐field imaging of microvascular velocity and diameter pulsatility, crucial for understanding the complex function of the pulsating vasculature, remains a challenge.

In this study, we present a new algorithm and method for robust full‐field characterization of microvascular pulsatility in the brain cortex. We use laser speckle contrast imaging to acquire blood flow images at ≈200 frames per second, analyze them to achieve sub‐frame sampling, and simultaneously extract pulsatility indexes (*PI*) for vessel diameter and blood flow. We complement LSCI data with Lomb–Scargle power spectrum analysis of capillary diameter dynamics obtained using two‐photon microscopy. We apply the combined techniques to assess cerebral perfusion and pulsatility in a longitudinal study of male wild‐type (C57BL/6JRj) mice from 18 to 81 weeks, and complement these findings with a cross‐sectional comparison between sexes. Furthermore, we also perform measurements on anesthetized mice at every timepoint using isoflurane and ketamine‐xylazine, the most commonly used anesthesia types.^[^ ^37^, ^38^ ^]^ Our findings reveal remarkably stable blood flow index (*BFI*) and vascular diameter (*D*) over the mouse lifespan. Pulsatility parameters are consistent with stable perfusion and show no change until the final weeks of observation, when an increase in arterial diameter pulsation appears. In contrast, measurements taken under anesthesia display a range of age‐dependent and age‐independent changes. Most strikingly, despite their distinct mechanisms and effects on the vasculature, isoflurane and ketamine‐xylazine significantly increase perfusion pulsatility and affect diameter pulsatility. While the effects of anesthesia on mean cerebral perfusion are well‐documented, their impact on the dynamic, pulsatile nature of microvascular flow remains largely unexplored. Our results highlight a previously overlooked detrimental effect of anesthesia when assessing cerebrovascular function, and also suggest that pulsatility can change dramatically depending on the subject's condition, providing a new approach to address the impact of microvascular pulsatility on brain health.

## 2 Results

### 2.1 Pulsatility and Perfusion in the Aging Brain

**Figure 1:**
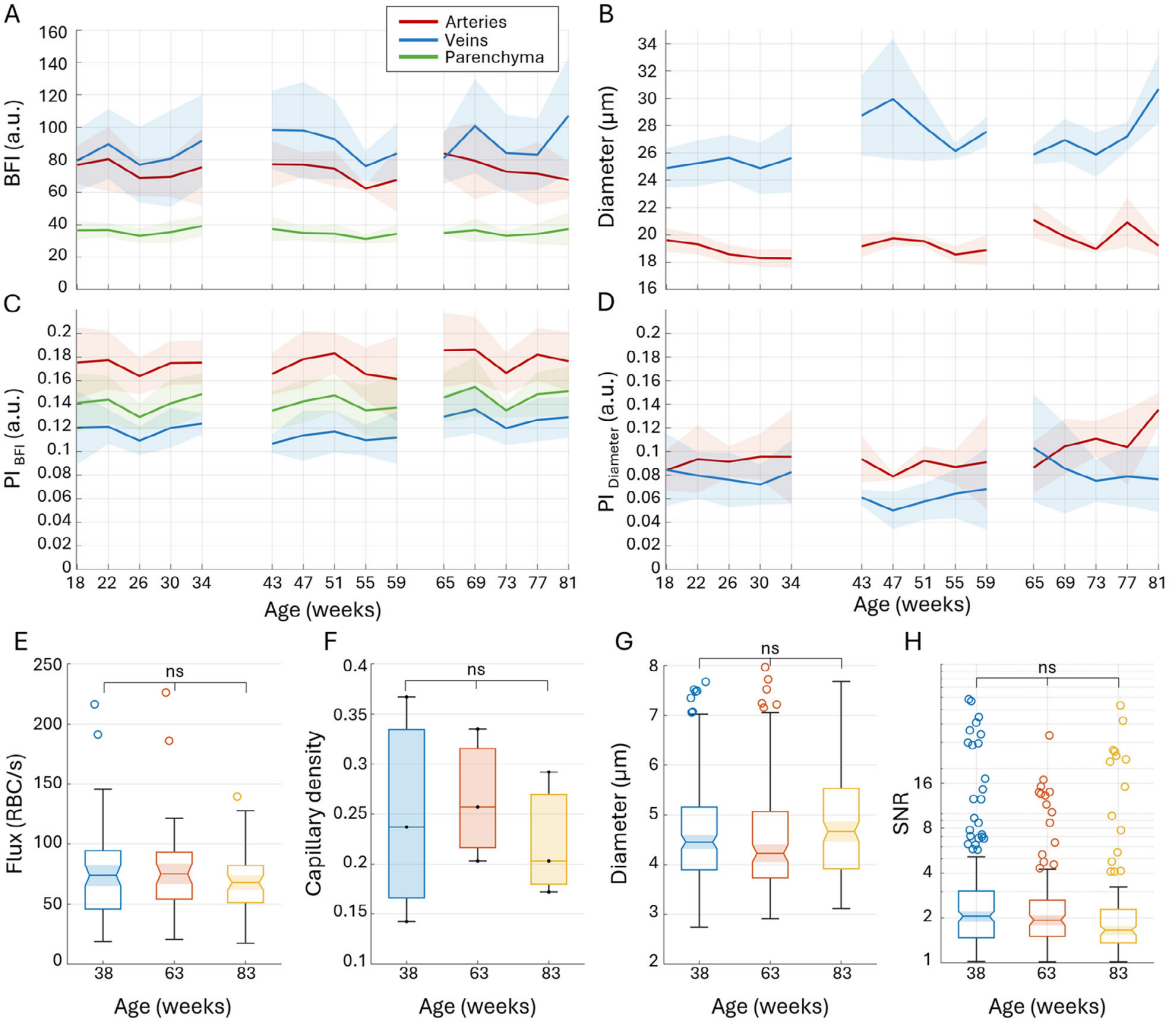
LSCI (n=16) A–D) and TPM (n=9) E–H) measurements of perfusion, diameter, and respective pulsatility in aging awake C57BL/6JRj mice. A ‐ average blood flow index (*BFI*) in small arteries (red), veins (blue), and parenchyma (green) corresponding to the mouse age. B ‐ average diameters (*D*) of arteries and veins. C ‐ average flow pulsatility index (*PI*_*BFI*_) for arteries, veins, and parenchyma. D ‐ average diameter pulsatility index (*PI*_*D*_) for arteries and veins. Solid lines and shaded areas reflect the mean and standard deviation for the corresponding measurements across animals. Details on the number of animals and vascular segments for each timepoint are provided in Supplementary Figure 1. Capillaries scanned at age 38 weeks (n=75), 63 weeks (n=51), and 83 weeks (n=63). E ‐ capillary flux, F ‐ capillary density, G ‐ capillary diameter, and H ‐ pulsatility signal‐to‐noise ratio as estimated from TPM data. The data are presented as mean ± SD, *P*‐values are calculated using one‐way ANOVA, *ns* indicate *P* > 0.05.

Microvascular perfusion and pulsatility measurements in aging awake mice are presented in **Figure** **1**. Surprisingly, *BFI* Figure 1A) and the pulsatility of blood flow (*PI*_*BFI*_) Figure 1C) almost do not change across the entire observation period, with variation in average values being below 5% for *BFI* and below 6% for *PI*_*BFI*_ between age groups (Figure 1C). It highlights the stability of the perfusion as opposed to age‐related perfusion decline hypothesized in some studies.^[^ ^39^, ^40^, ^41^ ^]^ The intra‐group variation between timepoints is also low, 10% for *BFI* and 6% for *PI*_*BFI*_, reflecting the robustness of the measurements. Average *BFI* and *PI*_*BFI*_ images, as well as statistics on the number of identified segments, are shown in Figures S1–S3 (Supporting Information), respectively. Notably, the *PI*_*BFI*_ of ≈0.17 in small arteries, ≈0.14 in the parenchyma, and ≈0.12 in small veins (Figure 1C) is not only age but also system‐independent (as a relative measurement, see Methods), which makes it a reference for healthy mice within the given age and vessel diameter. The *BFI* pulsatility significantly differs between all vascular regions (*p* < 0.0001). Diameter *D* and its pulsatility (*PI*_*D*_) measurements, shown in Figure 1,B, and D, follow almost the same pattern, except for the last timepoints, where the average venular diameter appears to increase after week 73. Interestingly, the average arterial diameter does not increase at the same time. Instead, arterial diameter pulsatility *PI*_*D*_ is significantly elevated from 0.09 at week 65 to 0.14 at week 81 (*p* = 0.00971). Such an observation could imply that arteries become less stiff with age. TPM measurements reveal no significant changes in capillary flux, density, or diameter (Figure 1E–G), which aligns well with the stable BFI throughout the observation period. The average capillary diameter also does not show a significant difference (Figure 1G), although the median diameter has increased at week 83, matching the venular diameter increase observed with LSCI.

**Figure 2:**
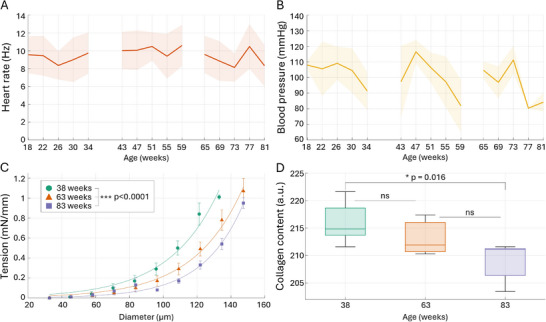
Systemic measurements A,B) in aging awake mice (n=16), as well as ex vivo data (n=16) C,D) for cerebrovascular changes over different ages. A ‐ average heart rate measured from the LSCI data, corresponding to the data shown in Figure 1. B ‐ average blood pressure from the tail‐cuff measurements at the end of each LSCI recording. In week 77, only one valid blood pressure measurement is available due to the malfunction of the cuff, resulting in a standard deviation of zero. C ‐ the passive inner diameter‐wall tension relationships, measured under isometric conditions for isolated middle cerebral arteries. Diameter‐tension curves for all age groups are significantly different, with *p* < 0.0001 for all group comparisons using the extra sum‐of‐squares F test. D ‐ histological measurements of collagen presence in the wall of the corresponding middle cerebral arteries. The data are presented as mean and ± SEM, *ns* indicate *P* > 0.05.

Systemic and ex vivo measurements align well with the perfusion and pulsatility imaging. The average heart rate remained within 8.1–10.6 Hz, with less than 7% variation between the average for the age groups and less than 9% intra‐group variation between the timepoints (**Figure** **2**A). Similarly, the average blood pressure has generally remained between 80.3 and 116.5 mmHg with inter‐group variations of <10% and intra‐group variations <15% (Figure 2B). Neither heart rate nor blood pressure changes could be associated with the increased venular diameter and arterial pulsatility observed in the last timepoints (Figure 1B,D). The passive diameter‐tension curves obtained with isometric wire‐myography of middle cerebral arteries confirm the late‐onset diameter pulsatility changes, showing increased vascular wall compliance with age (Figure 2C). The histology data further confirms it, showing a reduction in collagen with age (Figure 2D).

### 2.2 Pulsatility Along the Microvascular Network

**Figure 3:**
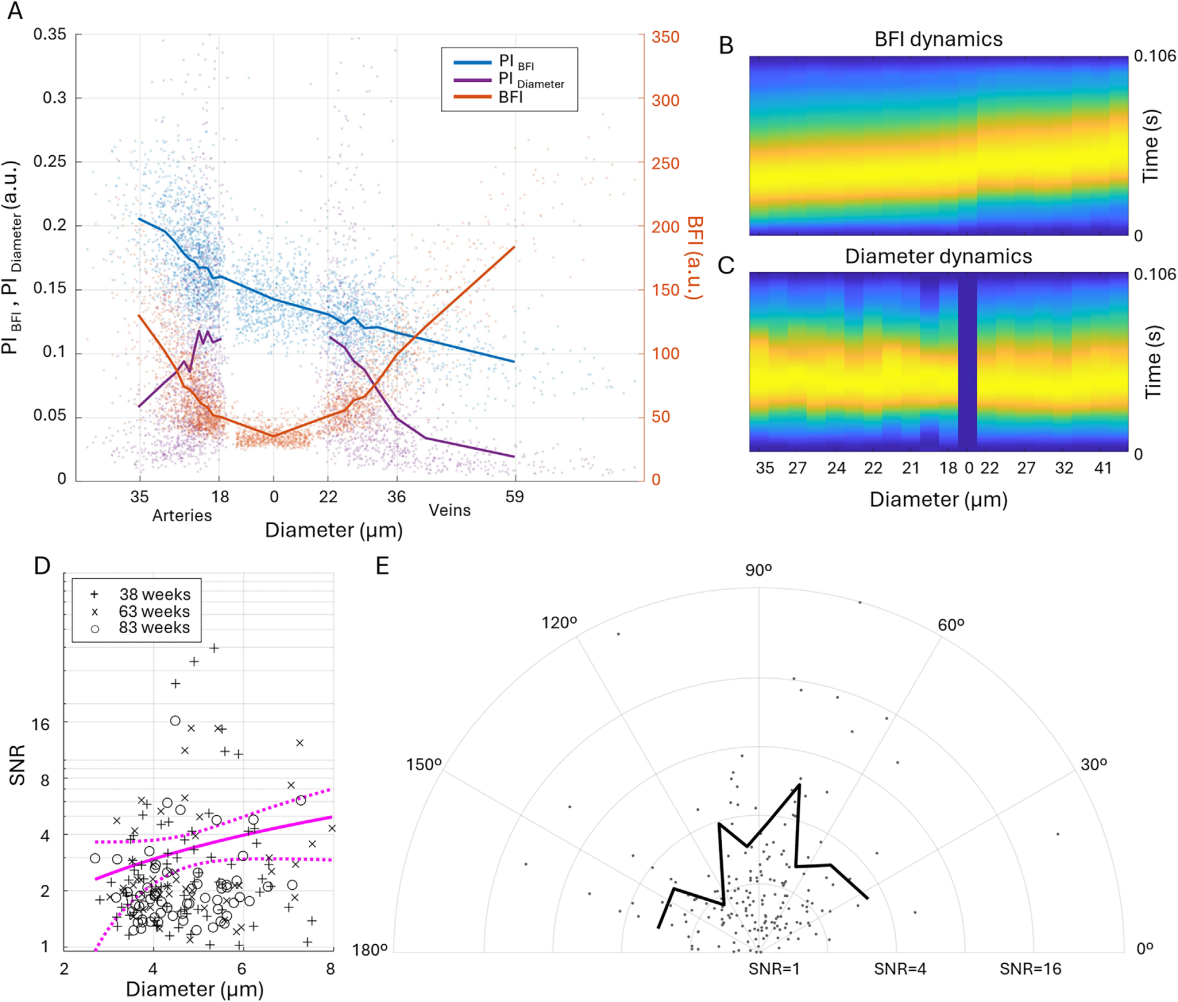
Changes in pulsatility and perfusion across the microvascular network for all measurements (n=16). A)–average diameter and perfusion pulsatilities *PI*_*D*_ and *PI*_*BFI*_ (yellow and blue lines, left Y‐axis), and blood flow index *BFI* (red, right Y‐axis) plotted as a function of the average diameter *D* across the imaged microvascular network. Points indicate individual measurements. Note that parenchymal measurements, by definition, do not have a diameter value associated with them ‐ the respective markers were assigned to a random diameter that is below the smallest vessel diameter for better visualization. B) ‐ the color‐coded shape of the BFI pulse. Color corresponds to the normalized BFI, changing from 0 (blue) to 1 (yellow). The Y‐axis reflects time, and the X‐axis is the same as in panel A, showing the diameter with arteries on the left and veins on the right. C) ‐ the color‐coded shape of the diameter pulse. D) ‐ log‐scale SNR at the cardiac frequency calculated from Lomb–Scargle power spectra of capillary diameters. Markers correspond to the measurements done at 38 (“+”), 63 (“x”), and 83 (“o”) weeks, respectively. Magenta lines show the linear regression model and 95% confidence intervals calculated across all measurements. E) ‐ a polar plot of capillary pulsatility SNR for different vessel orientations relative to the coronal plane estimated from TPM line scans. Points refer to individual capillaries, and the black line is the mean.

**Figure 4:**
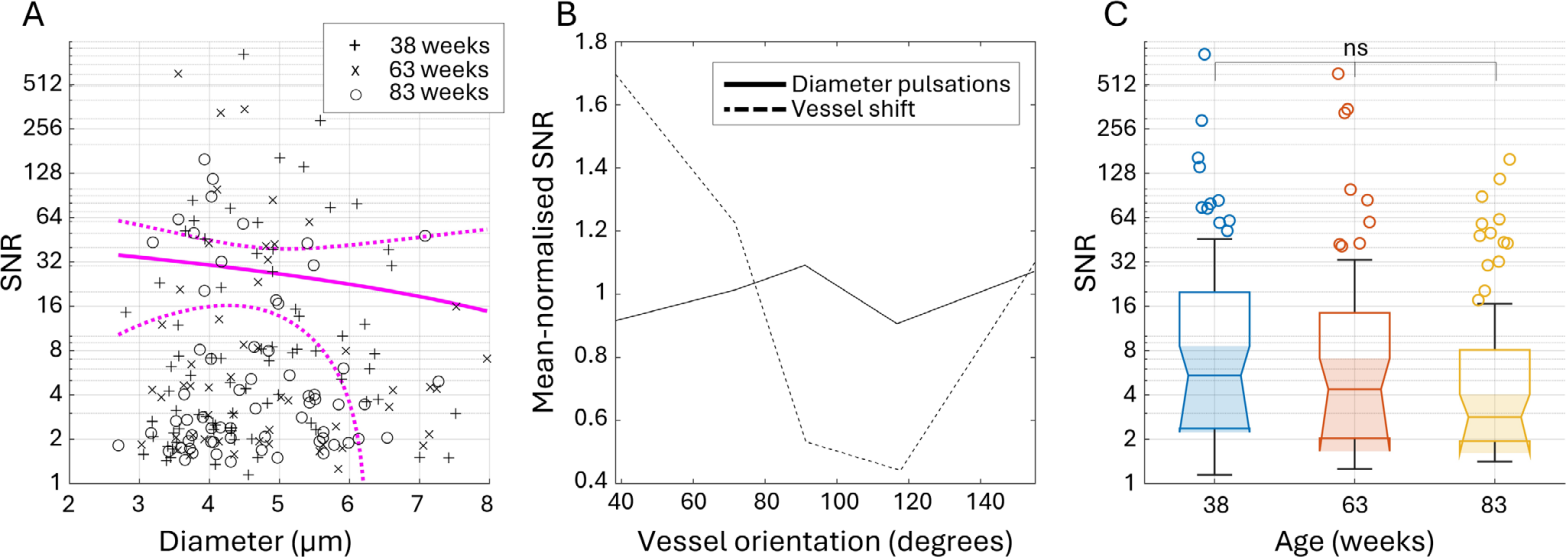
Lomb–Scargle analysis of capillary wall dynamics associated with the brain‐wide tissue displacement during the cardiac cycle. A) ‐ log‐scale SNR at the cardiac frequency calculated from Lomb–Scargle power spectra for the changes of capillary wall coordinates. Markers correspond to the measurements done at 38 (“+”), 63 (“x”), and 83 (“o”) weeks, respectively. Magenta lines show the linear regression model and 95% confidence intervals calculated across all measurements. B) ‐ mean‐normalized SNR of displacement and capillary pulsatility for different vessel orientations. Capillary orientation is grouped in 20°‐wide bins. C) ‐ displacement SNR grouped by animal age. The data are presented as mean ±*SD*, *P*‐values are calculated using one‐way ANOVA, *ns* indicate *P* > 0.05.

**Figure** **3** shows how pulsatility and perfusion change along the microvascular network, from arteries to veins across all ages. Naturally, the (*BFI*) displays an inverse bell shape, as it first reduces with decreasing diameter on the arterial side, reaching minimal values in the parenchyma and increasing along with the diameter on the venular side (Figure 3A–C). The *PI*_*BFI*_ is consistently decreasing, from as high as 0.21 in large arteries to as low as 0.09 in large veins, fitting the flux pulsatility decrease shown in Ref. [33]. It reflects how flow oscillations are dampened along the elastic vascular network. The diameter pulsatility displays behavior that is inverted compared to the BFI ‐ large arteries and veins appear as non‐pulsating (average *PI*_*D*_ < 0.06), while the smallest vessel experiences the most extensive changes in the diameter (average *PI*_*D*_ > 0.11). Furthermore, while the BFI pulse shape is consistently changing along the network, with the peak getting more Gaussian‐like and more delayed (Figure 3B), the diameter pulse shape remains unchanged (Figure 3C). More details on the exact correlations between LSCI measurements are provided in the supplementary Figure 4. Lomb–Scargle power spectrum analysis of the TPM data confirms the presence of the cardiac‐cycle‐associated diameter fluctuations in capillaries (Figure 3D,E). Linear regression of the signal‐to‐noise (SNR) ratio at the cardiac frequency shows that pulsatility increases with the diameter, similar to changes in the absolute pulse magnitude for small vessels measured with LSCI. Interestingly, both LSCI and TPM data show severe pulsatility heterogeneity, where the diameter oscillations in a minority of blood vessels are several times stronger than for the majority of them (Figure 3A,D). Furthermore, as shown in Figure 3E, the SNR is not correlated with vessel orientation, confirming that it is associated with vascular pressure wave propagation, rather than brain‐wide tissue displacement, as characterized in **Figure** **4**. The estimated SNR for vessel displacement is much higher than for capillary pulsatility and, unlike the latter, is strongly orientation‐dependent (Figure 4A,B). The displacement is most pronounced in vessels oriented at roughly 40 degrees to the coronal plane, which matches the expected orthogonal direction to the pressure wave traveling through the brain tissue from the circle of Willis (Figure 4B). Most interestingly, the displacement SNR also tends to decrease with age, although not statistically significant, possibly reflecting changes in tissue rigidity (Figure 4C).

### 2.3 Confounding Effects of Isoflurane

**Figure 5:**
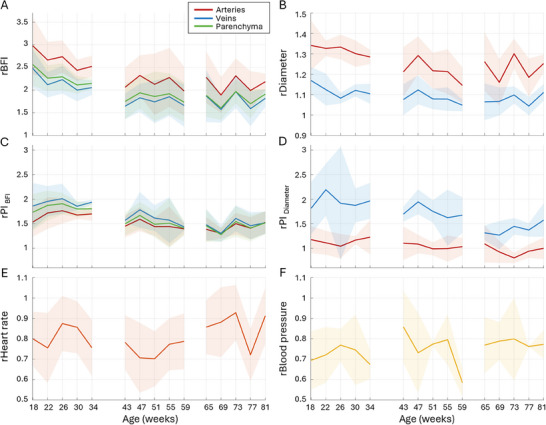
Age‐dependent effects of isoflurane on brain perfusion and pulsatility. The results are normalized by the respective measurements in the awake condition, with *p* − *values* calculated for anesthetized versus awake in each vessel type. A) ‐ average relative blood flow index change (*rBFI*) in small arteries (red), veins (blue), and parenchyma (green). *P* − *values* are *p* < 0.0001 for arteries, parenchyma, and veins when compared to awake data across all timepoints. B) ‐ average relative diameter (*rD*), with *p* < 0.0001 for arteries and *p* = 0.0016 for veins. C) ‐ average relative BFI pulsatility index change (*rPI*_*BFI*_), with *p* < 0.0001 in all regions. D) ‐ average relative diameter pulsatility index change (*rPI*_*D*_), with *p* = 0.9407 for arteries and *p* < 0.0001 for veins. E) ‐ average relative heart rate change (*p* < 0.0001). F) ‐ average relative blood pressure change (*p* < 0.0001). The data are presented as mean ± SD, *P*‐values are calculated using one‐way ANOVA, the exact *p* − *values* are reported, except for *p* < 0.0001.

Blood velocity and vessel diameter are known to be strongly affected by isoflurane anesthesia due to its potent vasodilatory action.^[^ ^42^ ^]^ **Figure** **5** provides further insight into the isoflurane‐induced changes in perfusion and pulsatility of aging mice. Data are presented normalized by the respective measurements in the awake condition. As expected, BFI and diameters increased significantly (*p* < 0.0001). Interestingly, the increase is age‐dependent ‐ it was the highest in 18‐week‐old mice, where the arterial BFI increased by 200% and the vascular diameters by 34% compared to awake mice. The effect weakened in older mice, reaching an increase of ≈100% and ≈20% for BFI and diameter, respectively, at 43 weeks (Figure 5A,B). *PI*_*BFI*_ has also significantly (*p* < 0.0001) increased, in line with the BFI changes (Figure 5C). Pulsatility increases the least in arteries and the most in veins ‐ for the youngest age group, the average relative pulsatility in BFI (*rPI*_*BFI*_) is 1.7 in arteries, 1.8 in the parenchyma, and 1.9 in veins. The tendency is the same, albeit less pronounced, for the second and the third age groups, with the respective average *rPI*_*BFI*_ of 1.5 and 1.42 in arteries, 1.51 and 1.44 in the parenchyma, and 1.6 and 1.47 in veins. The diameter pulsatility index (Figure 5D), on the other hand, has only significantly increased in veins *p* < 0.0001, with the average *rPI*_*D*_ being ≈1.95, ≈1.7, and ≈1.4 in the respective age groups. Arterial *PI*_*D*_ experienced only a minor increase ‐ ≈14% for the first age group, ≈4% in the second group, and even decreased ≈5% in the oldest group. Heart rate and blood pressure (Figure 5E,F) decreased by 20 − 25% compared to the awake condition but did not display age‐dependent dynamics. Figure S6A,C,D (Supporting Information) provides more details on how isoflurane affects the pulsatility along the microvascular network.

### 2.4 Confoundingc effects of Ketamine‐Xylazine

**Figure 6:**
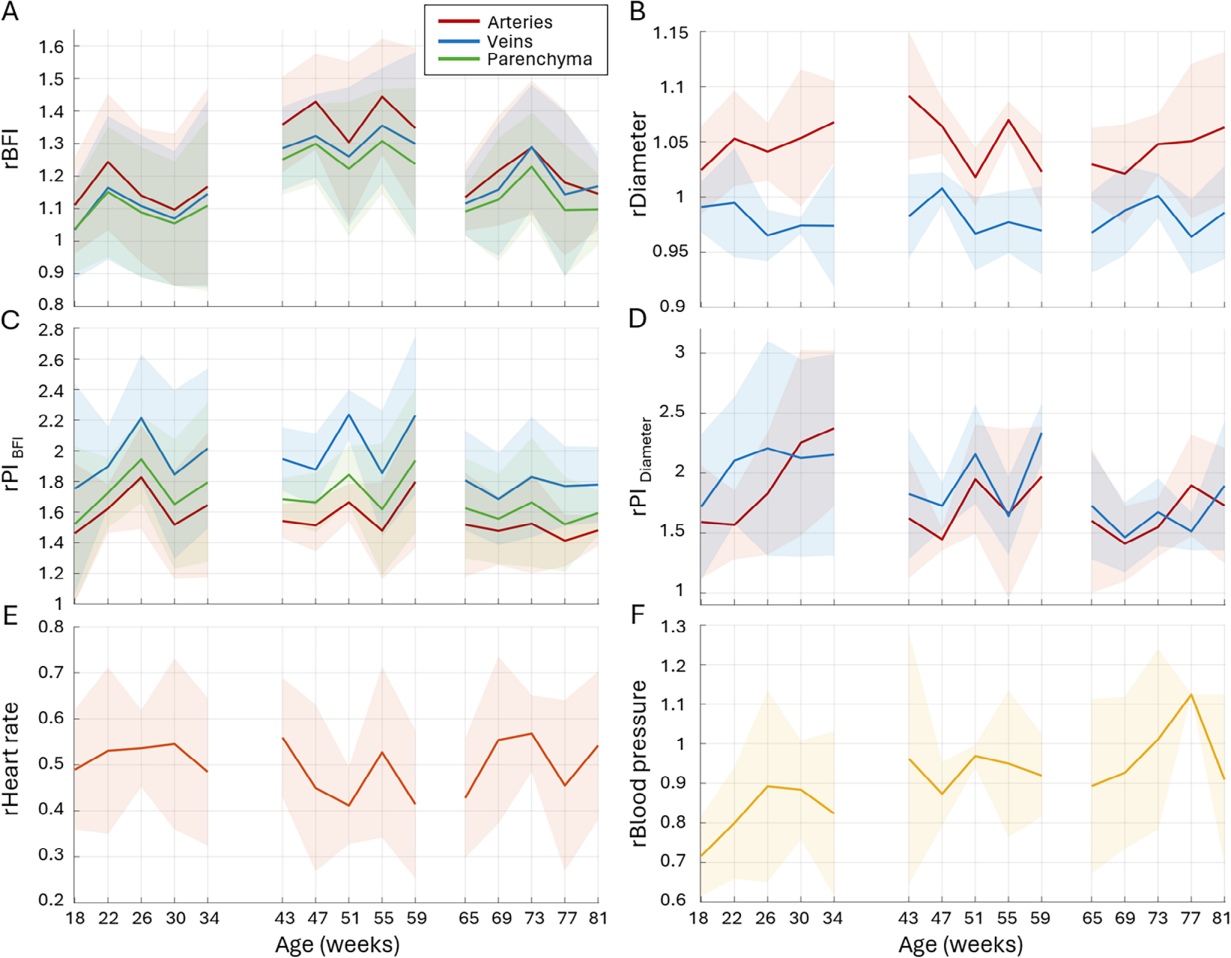
Effects of ketamine‐xylazine anesthesia on brain perfusion and pulsatility. The results are normalized by the respective measurements in the awake condition, with *p* − *values* calculated for anesthetized versus awake conditions in each vessel type. A) ‐ average relative blood flow index change (*rBFI*) in small arteries (red), veins (blue), and parenchyma (green). Respective *p* − *values* when comparing to awake data across all timepoints are *p* = 0.0117 for arteries, *p* = 0.0414 for parenchyma, and *p* = 0.030 for veins. B) ‐ average relative diameter (*rD*), with the respective *p* − *values* *p* = 0.0422 for arteries and *p* = 0.7672 for veins. C) ‐ average relative BFI pulsatility index change (*rPI*_*BFI*_), with *p* < 0.0001 in all regions. D) ‐ average relative diameter pulsatility index change (*rPI*_*D*_), with the respective *p* < 0.0001 for arteries and veins. E) ‐ average relative heart rate change (*p* < 0.0001). F) ‐ average relative blood pressure change (*p* = 0.0337). The data are presented as mean ± SD, *P*‐values are calculated using one‐way ANOVA, the exact *p* − *values* are reported, except for *p* < 0.0001.

Like isoflurane, the ketamine‐xylazine combination is widely used in small animal research as an anesthetic and an immobilizing agent when doing in vivo imaging.^[^ ^43^ ^]^ Unlike isoflurane, however, ketamine‐xylazine does not have a vasodilatory effect and affects the perfusion less, leading to the *BFI* increase of 10–40% (**Figure** **6**A) and minor‐to‐no differences in vascular diameter (Figure 6B) without a clear age‐associated pattern. Despite that, ketamine‐xylazine has caused an even more profound increase in pulsatility compared to isoflurane ‐ flow pulsatility has, on average, increased by ≈40 − 80% in arteries, ≈50 − 90% in the parenchyma, and ≈75 − 120% in veins. The diameter pulsatility has also risen, with the average *PI*_*D*_ increasing by 50–160% in both arteries and veins. The dramatic changes in pulsatility are accompanied by a two‐fold decrease in the heart rate (Figure 6E) and subtle changes in blood pressure (Figure 6F). The latter remained generally similar to the awake condition, although it appears to decrease ≈20% in the youngest age group. More details on how the ketamine‐xylazine combination affects the pulsatility along the microvascular network are provided in Figure S6B,E,F (Supporting Information).

### 2.5 Sex‐Related Differences in Microvascular Dynamics

**Figure 7:**
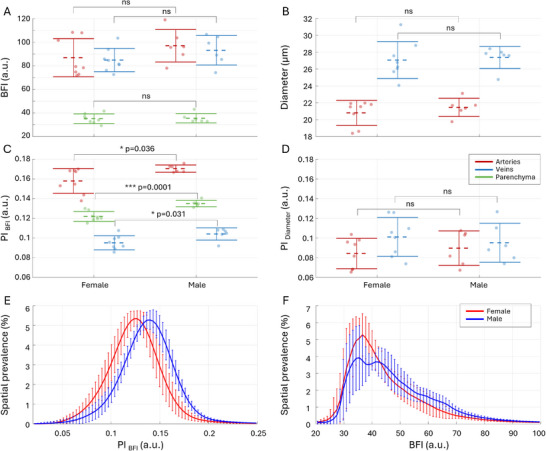
Sex‐dependent differences in cerebral microvascular dynamics assessed with LSCI in 50‐51 week‐old female (*n* = 8) and male (*n* = 6) C57BL/6JRj mice. A) ‐ average blood flow index (*BFI*) in small arteries (red), veins (blue), and parenchyma (green) in females and males of the same age (50–51 weeks). B)‐ average diameters (*D*) of segmented arteries and veins. C) ‐ average flow pulsatility index (*PI*_*BFI*_) for arteries, veins, and parenchyma. D) ‐ average diameter pulsatility (*PI*_*D*_) for arteries and veins. The dashed line and the error bars reflect the mean and the standard deviation. E) ‐ spatial prevalence of stratified *PI*_*BFI*_ values in females and males. F) ‐ spatial prevalence of stratified *BFI* values in females and males. The data are presented as mean ± SD, *P*‐values are calculated using one‐way ANOVA, the exact *p* − *values* are reported, except for *p* < 0.0001, *ns* indicate *P* > 0.05.

To assess sex‐related differences in microvascular dynamics, we performed an additional set of experiments and compared key hemodynamic parameters in a cohort of 50–51 week‐old female and male mice. Mean perfusion, as measured by *BFI*, and average vessel diameters did not differ significantly between sexes across arteries, veins, or parenchyma (**Figure** **7**A,B). In stark contrast, the flow pulsatility index (*PI*_*BFI*_) was significantly higher in males than in females across all measured compartments: arteries (*p* = 0.036), veins (*p* = 0.031), and parenchyma (*p* = 0.0001) (Figure 7C). This difference was specific to flow dynamics, as no significant sex‐related variation was observed in diameter pulsatility (*PI*_*D*_) (Figure 7D). The spatial prevalence distributions further corroborate these findings, illustrating similar BFI profiles but distinctly separate *PI*_*BFI*_ profiles between sexes (Figure 7E,F) across the entire microvascular network.

## 3 Discussion

In the present study, we propose a novel, highly robust approach for simultaneously assessing microvascular flow and diameter pulsatility using full‐field blood flow imaging and two‐photon microscopy, and apply it to advance our understanding of cerebral hemodynamics in aging awake and anesthetized mice. Our results contribute to the growing body of studies suggesting that there is no age‐related perfusion decrease^[^ ^44^, ^45^ ^]^ in the cortex, at least in the awake male C57BL/6JRj mice younger than 81 weeks (Figure 1A). While there are reports supporting an age‐related perfusion decline^[^ ^39^, ^40^, ^41^ ^]^ or even a slight increase,^[^ ^46^ ^]^ we hypothesize that these discrepancies are likely a consequence of different animal preparations, perfusion quantification methodology, and, crucially, the use of anesthesia. The absence of age‐dependent changes in the heart rate, blood pressure, capillary flux, density, and diameters supports the observed perfusion stability. We show that microvascular BFI pulsatility is similarly stable and reaches 12–18% (Figure 1C), becoming comparable in magnitude with hyperaemic responses observed during functional stimulation.^[^ ^47^ ^]^ Such stability can be seen as either an absence of vascular changes or a complex combination of factors that balance each other, thereby maintaining stable cerebral perfusion. The latter is more probable due to the previously confirmed functional vascular and neurovascular abnormalities in the aging mouse brain.^[^ ^47^, ^48^ ^]^ Our results suggest that pulsatility values along the vascular tree depend on the subject's condition, not limited to perfusion changes, as observed in animals under anesthesia (Figure S6, Supporting Information). More specifically, a higher *PI*_*BFI*_ and the slowdown of its decrease can be associated with increased arterial stiffness or hemodynamic resistance (e.g., due to capillary rarefaction).^[^ ^13^, ^49^ ^]^ A decrease in *PI*_*BFI*_ and, possibly, a faster decline can be associated with upstream occlusion or flow reduction (e.g., in stroke or transient ischemia).^[^ ^50^ ^]^

The diameter and its pulsatility align with the *BFI* stability until week 73. At later ages, the average vein diameter increases significantly (Figure 1B). It is accompanied by a non‐significant but noticeable increase in the median capillary diameter (Figure 1G) and a decrease in capillary pulsatility (Figure 1H). Surprisingly, this change is not reflected in arterial diameter or *BFI* in arteries or parenchyma. Instead, average arterial diameter pulsatility *PI*_*D*_ during week 81 becomes significantly higher than in week 65 (Figure 1D). It can imply either a stronger pulse reaching the brain, the cerebral arteries becoming less stiff with age, or both. A stronger pulse to reach the brain requires either an increased cardiac output, which is unlikely, or increased stiffness of the large arteries, such as the aorta. While little is known about vascular stiffness in aging mice, there are reports of a mild increase in aorta stiffness by the age of 80 weeks^[^ ^24^, ^51^, ^52^ ^]^ under anesthesia. Contrary to the increase in aortic stiffness, our findings from isometric wire myography show that middle cerebral arteries become more compliant with age (Figure 2C). The histology data show a decrease in collagen deposition on the cerebrovascular wall (Figure 2D) and a tendency for a reduction in the arterial wall thickness, which supports the myography data. Small arteries becoming less stiff and allowing stronger diameter pulsatility at a later age may hint at the mechanism countering increased flow pulsatility caused by stiffer large vessels, therefore protecting the brain's capillaries.

Aside from the changes at a late age, the distribution of the average *PI*_*D*_ across the microvascular network is particularly interesting (Figure 3A). It starts low (average *PI*_*D*_ < 0.05) in arteries larger than 30µ*m* in diameter and then increases for smaller vessels, reaching the peak of *PI*_*D*_ > 0.1 in the smallest resolvable arterioles and venules with ≈12µ*m* diameter, and then decreases to its lowest values in larger veins. Together with the rapid decrease in the *PI*_*BFI*_ in arteries below ≈12µ*m* diameter, it suggests that these small vessels play a prominent role in microvascular pulse dissipation and are likely candidates for the “drivers” of pulse‐associated fluid motion.^[^ ^22^ ^]^ Crucially, the diameter pulsatility appears to be strongly heterogeneous, with both LSCI and TPM data showing that at the same average diameter, some vessels pulsate several times more than others. Understanding the cause of heterogeneity requires further exploration, but it may be related to the tissue's structural properties in the proximity of the vessels or the local tone of mural cells, including pericytes. At the same time, it highlights that some of the small vessels might be especially vulnerable to abnormal increases in pulsatility, which are likely to disrupt the blood‐brain barrier.^[^ ^15^ ^]^

Pulsatility and perfusion may significantly change in later ages, closer to 105–120 weeks of age, corresponding to the maximum life expectancy of the C57BL/6 male mice.^[^ ^53^ ^]^ In such a perspective, 81 weeks “correspond” to only 60 human years, at which cerebral perfusion decline may not be present.^[^ ^54^ ^]^ At this age, mice also show almost no signs of “natural” cognitive decline,^[^ ^55^ ^]^ similar to humans, where these signs before 65 years are uncommon and are referred to as “early‐onset” dementia.^[^ ^56^ ^]^ Assuming perfusion and cognition change in older animals suggests that the diameter pulsatility changes we observed at week 81 might precede other abnormalities, making their further exploration pivotal for understanding the age‐related brain perfusion changes and associated pathologies. While no animal model perfectly recapitulates human ageing, the C57BL/6 mouse is a widely accepted model that exhibits key features of age‐related vascular changes, including a gradual increase in aortic stiffness.^[^ ^51^, ^52^ ^]^ The fundamental biophysical principles governing pulse wave propagation and damping are conserved across mammals. Therefore, our findings on how pulsatility is managed at the microvascular level in mice provide crucial mechanistic insights into processes relevant to human health. For instance, the pathologically high pulsatility we induced with anesthesia mirrors the hemodynamic state in humans with significant arterial stiffness, a major risk factor for cerebral small vessel disease, white matter hyperintensities, and cognitive decline.^[^ ^8^, ^13^ ^]^

Although known to have multiple systemic and local effects, general anesthesia is still broadly applied in animal research, either in settings where an awake animal preparation is impossible or to save the required effort. Isoflurane and ketamine‐xylazine are, in particular, among the most common anesthesia choices.^[^ ^57^ ^]^ Isoflurane is a general inhalation anesthetic that conveniently allows long‐term stability when administered via a mask or intubation. However, as an agonist of adenosine triphosphate‐sensitive K^+^ channels, isoflurane causes hyperpolarisation of the smooth muscle cells and, therefore, potent vasodilation,^[^ ^58^, ^59^ ^]^ raising concerns for the interpretation of the perfusion measurements and functional responses. Our results show that induction of 1.5% isoflurane via a non‐airtight mask can cause an increase of up to 200% in BFI and 34% in arterial diameter in young mice (Figure 5A,B). Most critically, we show that the isoflurane‐associated increase in perfusion and diameters strongly weakens with age (by ≈60%), as was indirectly reported before.^[^ ^60^ ^]^ Such age‐dependent effects would make any aging‐related studies challenging to interpret, especially considering that heart rate and blood pressure remain age‐independent. It might also be one of the key reasons for previous reports of cerebral blood flow decline in aging mice.^[^ ^39^, ^40^, ^41^ ^]^ The ketamine‐xylazine combination, unlike isoflurane, allows for simple injection routes, does not require gas induction equipment, and has less pronounced effects on the brain vasculature and perfusion. Our results also support the latter, demonstrating a stable vessel diameter and <40% increase in blood flow under ketamine‐xylazine anesthesia compared to awake (Figure 6A,B). Nevertheless, both ketamine and xylazine can cause systemic changes, which will inadvertently affect brain perfusion. Ketamine has a sympathomimetic effect, where it increases heart rate and blood pressure,^[^ ^61^ ^]^ whereas xylazine's action causes cardiovascular depression, including bradycardia,^[^ ^62^ ^]^ hypotension, and decreased cardiac output.^[^ ^63^ ^]^ The systemic effects, especially from slower and longer‐lasting xylazine, are reflected in the heart rate measurements, displaying a 50.6% reduction compared to the awake condition. Unlike isoflurane, however, ketamine‐xylazine has shown no explicit age‐dependent perfusion dynamics, possibly making it more optimal for aging studies requiring anesthesia.

We have addressed how anesthesia affects microvascular pulsatility in the brain cortex. The perfusion pulsatility index *PI*_*BFI*_ has significantly increased for both isoflurane and ketamine‐xylazine anesthesia despite their distinct effects on the perfusion. It follows the decline in age‐dependent dynamics in the case of isoflurane anesthesia (Figure 5C,D) and a stable elevation in all age groups for ketamine‐xylazine anesthesia (Figure 6C,D). Notably, the effect differs depending on the vessel type ‐ the *PI*_*BFI*_ increased relatively less in arteries than in veins. It may reflect arteries reaching the capacity limit to compensate for perfusion fluctuations, allowing a disproportionately stronger pulse to reach capillaries and veins. Such behavior can occur in pathologies where the vascular network's pulse dissipation ability is compromised, e.g., due to increased stiffness or elevated pulsation. Counterintuitively, it is not necessarily associated with vasoconstriction but can also be caused by extensive vasodilation, as confirmed by the diameter pulsatility changes under anesthesia. Accordingly, under isoflurane anesthesia, only venular *PI*_*D*_ is increased (by 20 − 120% depending on age, while for ketamine‐xylazine anesthesia, both arterial and venular *PI*_*D*_ are nearly doubled. We suggest a straightforward explanation of such dynamics. Isoflurane causes potent dilation of arteries, rendering them unable to change their diameter and dissipate the pulse. Thus, the increased pulsatile flow, reflected in higher *BFI* and *PI*_*BFI*_, propagates through the arterial side and reaches the veins at levels comparable to those of arterial pulsatility in healthy awake mice, causing strong diameter pulsations. Ketamine‐xylazine does not dilate arteries, but the heart rate is reduced to half of its value in awake mice while the *BFI* is maintained. That is only possible if every heartbeat becomes stronger, causing increased pulsatility. Unlike isoflurane, however, the cerebral arteries in ketamine‐xylazine anesthetized mice are not dilated and, thus, to some degree, can compensate for the perfusion fluctuations with passive diameter changes as reflected in arterial *PI*_*D*_ and *PI*_*BFI*_. That is, for ketamine‐xylazine anesthesia, a relative increase in parenchymal pulsatility is closer to the arteries, while for isoflurane, the change in parenchymal pulsatility is closer to the venular side. Nevertheless, even for mice under ketamine‐xylazine anesthesia, the cerebral arteries seem to reach the limit of how much they can compensate for perfusion fluctuations, as the pulsation strongly propagates to the venular side. A central and novel contribution of our study is the demonstration that general anesthesia, regardless of the specific agent's primary effect on vascular tone or heart rate, profoundly amplifies microvascular pulsatility in the brain. While the vasodilatory effects of isoflurane and the complex systemic effects of ketamine‐xylazine on mean perfusion are known, the near doubling of flow pulsatility is a previously uncharacterized phenomenon. This finding carries significant implications for the interpretation of a vast body of preclinical literature. Pathologically elevated pulsatility is linked to BBB disruption and microvascular damage.^[^ ^5^, ^6^ ^]^ Therefore, studies investigating these phenomena under anesthesia may have been conducted against a baseline of non‐physiological, high‐pulsatility hemodynamics, which could potentially confound the interpretation of experimental outcomes. Our results urge a re‐evaluation of anesthesia's role not merely as a tool for immobilization, but as an active modulator of cerebrovascular dynamics, which could also augment pulse‐associated vascular motion, disrupt blood‐brain barrier integrity by simultaneously dilating vessels and increasing intravascular pressure fluctuations, and alter functional responses, for example, by activating Piezo1 receptors^[^ ^64^, ^65^ ^]^). It suggests the importance of pulsatility effects when interpreting the results obtained under anesthesia, but also suggests a potential mechanism for anesthesia to improve drug delivery across the blood‐brain barrier.^[^ ^66^ ^]^ These findings may also have important translational implications for clinical neuroimaging. Sedation or general anesthesia is often required for brain scans or during intraoperative procedures in vulnerable populations, including children and patients with stroke or dementia. Our results, demonstrating that common anesthetic agents can double microvascular pulsatility, suggest that clinical perfusion or functional imaging under sedation may be conducted in a profoundly altered hemodynamic state, potentially leading to complex effects on quantitative measurements and patient recovery.

Our cross‐sectional analysis in the additional cohort of 50‐51 weeks old mice revealed clear sex‐specific differences in cerebral microvascular dynamics. While baseline perfusion (BFI) and vessel diameters were comparable between sexes (Figure 7A,B), the flow pulsatility index (*PI*_*BFI*_) was consistently and significantly lower in females across all vascular compartments (Figure 7C). This dissociation from mean flow highlights the specificity of pulsatility as a distinct vascular biomarker, capable of revealing hemodynamic characteristics not captured by traditional perfusion metrics. We propose that pulsatility is an emergent trait reflecting local biomechanical properties and upstream vascular behavior rather than just a direct marker of flow. These findings likely indicate sex‐dependent variations in cerebrovascular compliance and wave transmission characteristics, consistent with systemic vascular differences previously reported.^[^ ^67^ ^]^ Sex differences in pulsatility may originate from endothelial, hormonal, and structural vascular differences. Estrogen has demonstrated the ability to upregulate nitric oxide synthase activity, improve endothelial‐dependent vasodilation, and sustain compliance in both large and small vessels, thereby offering a protective pulsatile buffer in premenopausal females.^[^ ^68^ ^]^ Another possible contributor to sex‐dependent pulsatility variations is the differences in size and function of the heart,^[^ ^69^ ^]^ which therefore links heart and brain health. Such observations are clinically noteworthy, as excessive microvascular pulsatility has been associated with white matter damage,^[^ ^50^ ^]^ disruption of the blood–brain barrier, and the pathophysiology of Alzheimer's disease.^[^ ^70^ ^]^ Notably, recent sex‐stratified studies suggest that the influence of cerebral pulsatility on white matter lesion burden and cognitive decline may be more pronounced in females,^[^ ^71^ ^]^ despite absolute pulsatility being higher in males during early life. Consequently, our findings may represent early‐stage sex‐specific vulnerability trajectories, with distinct pathophysiological implications throughout the lifespan. Importantly, the blood flow homogeneity across sexes in our study highlights the specificity of pulsatility as a distinct vascular biomarker. This suggests that pulsatility is not just a marker of perfusion pressure or flow, but an emergent trait of local biomechanical conditions and the behavior of upstream vessels.

Aside from providing valuable physiological insights, the obtained results validate the robustness of the proposed method. The optimized design of the LSCI system we developed (see Experimental Section for details) allowed average measurements with less than 10% intra‐group variation and less than 10% intra‐animal variation across all timepoints. Considering physiological variability in perfusion, which undeniably contributed to the measurement variation, it confirms that the system provides reliable absolute BFI measurements and excels in longitudinal studies or studies comparing perfusion in different animal models. It can be straightforwardly integrated as part of other imaging systems, e.g., multi‐photon microscopy or optical coherence tomography, and provides unparalleled full‐field, high frame‐rate insight into vascular and perfusion dynamics simultaneously. Crucially, it achieves this at a microscopic resolution, enabling the characterization of pulsatility dynamics along the entire microvascular tree, from resistance arterioles to capillaries, a feat not possible with current clinical or preclinical MRI and ultrasound technologies. The pulsatility measurements are inherently normalized and, therefore, system‐independent, allowing for direct comparison between studies. This enables the use of the presented *PI*_*BFI*_ and *PI*_*D*_ as a reference in healthy aging wild‐type mice. The TPM and ex vivo data align well with the LSCI, supporting our observations.

Nevertheless, we must acknowledge several limitations of the study and considerations for future research. First and foremost, despite becoming the gold standard for optical brain imaging, chronically installed cranial windows might affect the tissue underneath, and the brain as a whole.^[^ ^72^ ^]^ Therefore, further research is required to understand how chronic cranial windows might alter vascular stiffness, pulsatility, and perfusion, and whether the interpretation of our results has to be changed accordingly. Moreover, it is possible that the ketamine‐xylazine effect on cerebrovascular pulsatility is dominated by xylazine, and a more thorough independent investigation of ketamine and xylazine actions is needed to understand their impact on microvascular pulsatility. From the imaging perspective, a larger field of view and higher optical resolution would improve the accuracy and precision of the results. We deliberately limited the field of view to 1024 × 512 pixels as a trade‐off to reduce the data volume. When not limited by the storage capacity, the field of view can be increased by up to 8 times (2048 × 2048 pixels) for a similar consumer‐grade CMOS sensor while keeping ≈200 Hz framerate. In such a case, by changing the objective, one could record more vessels at a higher resolution, getting more reliable data on small vessels. The latter is directly related to the limitations of the segmentation algorithm, as the diameter estimation error will naturally increase for pixel‐wise smaller vessels.^[^ ^73^, ^74^, ^75^, ^76^ ^]^ Furthermore, our optical imaging approach is restricted to the superficial cortex and does not provide information on hemodynamics in deep brain structures. Finally, at this step, we provide limited insight into sex‐dependent differences in microvascular perfusion, focusing on observing longitudinal changes only in male mice. Nevertheless, we have demonstrated that perfusion pulsatility is significantly reduced in females at the age of 50‐51 weeks, which calls for future studies to incorporate longitudinal monitoring of microvascular pulsatility in female mice, accounting for the estrous cycle and the transition to reproductive senescence. Despite the abovementioned limitations, we see the presented results and methods as seminal to future research on microvascular pulsatility and understanding its role in brain function.

## 4 Experimental Section

### Ethical Approval Declarations

All experimental protocols were approved by the Danish National Animals Experiments Inspectorate (permit no.: 2023‐15‐0201‐01399) and conducted according to the respective guidelines and the Directive 2010/63/EU of the European Parliament on the protection of animals used for scientific purposes and reported by the ARRIVE guidelines (Animal Research: Reporting In Vivo Experiments).^[^ ^77^ ^]^

### Animal Preparation

In total, data from 16 male C57BL/6JRj mice (Janvier, France) in three age groups, starting at 18 (n = 6), 43 (n = 4), and 65 (n = 6) weeks, were used for the ageing part of this study, and 50‐51 weeks old female (n = 8) and male (n = 6) for determining sex differences. All mice were acclimatized upon delivery in their cages for at least seven days and underwent surgical installation of a chronic cortical window over the left barrel cortex. For surgical procedures, mice were anesthetized in an induction chamber with 3% isoflurane in 100% oxygen and reduced to 1.5–2% isoflurane, depending on the individual mouse's response to the anesthesia, once moved to a homeothermic heating pad to maintain core body temperature at 37°C. Before surgery, analgesic (Buprenorphine, 0.1 mg kg^−1^), anti‐inflammatory (Carprofen, 5 mg kg^−1^), antibiotic (Ampicillin, 200 mg kg^−1^), and edema‐reducing corticosteroid (Dexamethasone, 4.8 mg kg^−1^) were administered intraperitoneally according to body weight. All fur was removed from the scalp with a rodent hair trimmer, followed by Veet hair removal cream. Local anesthesia (Xylocaine, 2 mg mL^−1^) was injected subcutaneously at the surgical site after the removal of the fur. A small droplet‐shaped section of skin was removed from the skull, and the edges were secured to the bone with tissue adhesive (3M Vetbond). Using a scalpel (blade #15), the periosteum was scraped off the exposed skull, and the skull was scored to increase surface area (blade #12) and, thus, to decrease the risk of headbar detachment. With a 0.05 mm diamond drill bur, a craniectomy was slowly performed without damaging the dura, including several breaks to avoid overheating the brain. The exact placement was determined based on skull size and age, but ≈1.5–2 mm AP, 3 mm ML to Bregma. If any bleeds resulted from the craniectomy, these were stopped with a sterile saline‐soaked hemostatic sponge before the placement of the glass window. Once the skull was thinned around the edge of the window, it was softened with a saline‐soaked hemostatic sponge for a few minutes before the skull piece was flipped off the brain. The brain was “wiped” clean with a saline‐soaked hemostatic sponge to remove as many bone particles as possible to reduce bone regrowth. A small drop of Kwik–Sil adhesive (World Precision Instruments, Inc.) was placed on the exposed brain, and an optically transparent round glass coverslip, Ø 4mm in diameter, was placed onto the window and kept in place with a toothpick attached to the stereotaxic frame and fixed to the skull using Loctite super glue gel, covering the edge of the glass and the skull. The entire skull, except for the glass, was then covered with Scotchbond Universal adhesive and cured with UV light. To fix the mouse during imaging sessions, a metal head plate was glued to the skull with light‐cured Relyx dental cement (3M). The entire skull was then covered with Relyx dental cement (3M) and cured to seal the surgical site and fix the head plate.

After a successful surgery, the mouse was placed in a heated recovery chamber and then returned to a new, clean cage. During the recovery period, which lasted a minimum of 10 days, weight and behavior were closely monitored. The mice received pain relief medication (Buprenorphine, Carprofen), antibiotic (Ampicillin), and fluids (saline) IP for four days following surgery, as well as soft food at the bottom of the cage to encourage weight gain. Following the recovery period, the awake mice were habituated to restraint and trained in a mock LSCI environment to minimize motion artifacts and stress during imaging. The habituation lasted for ten days, starting with 15 min of restraint and increasing by 15‐min increments daily until reaching 2 h. The mice were rewarded with sweetened condensed milk during the habituation. At all times, mice were housed in a 12 h light/dark cycle, 22‐24°C, 55 ± 10% humidity, with ad libitum access to food and water.

### Laser Speckle Contrast Imaging of Microvascular Pulsatility—Experimental protocol

**Figure 8:**
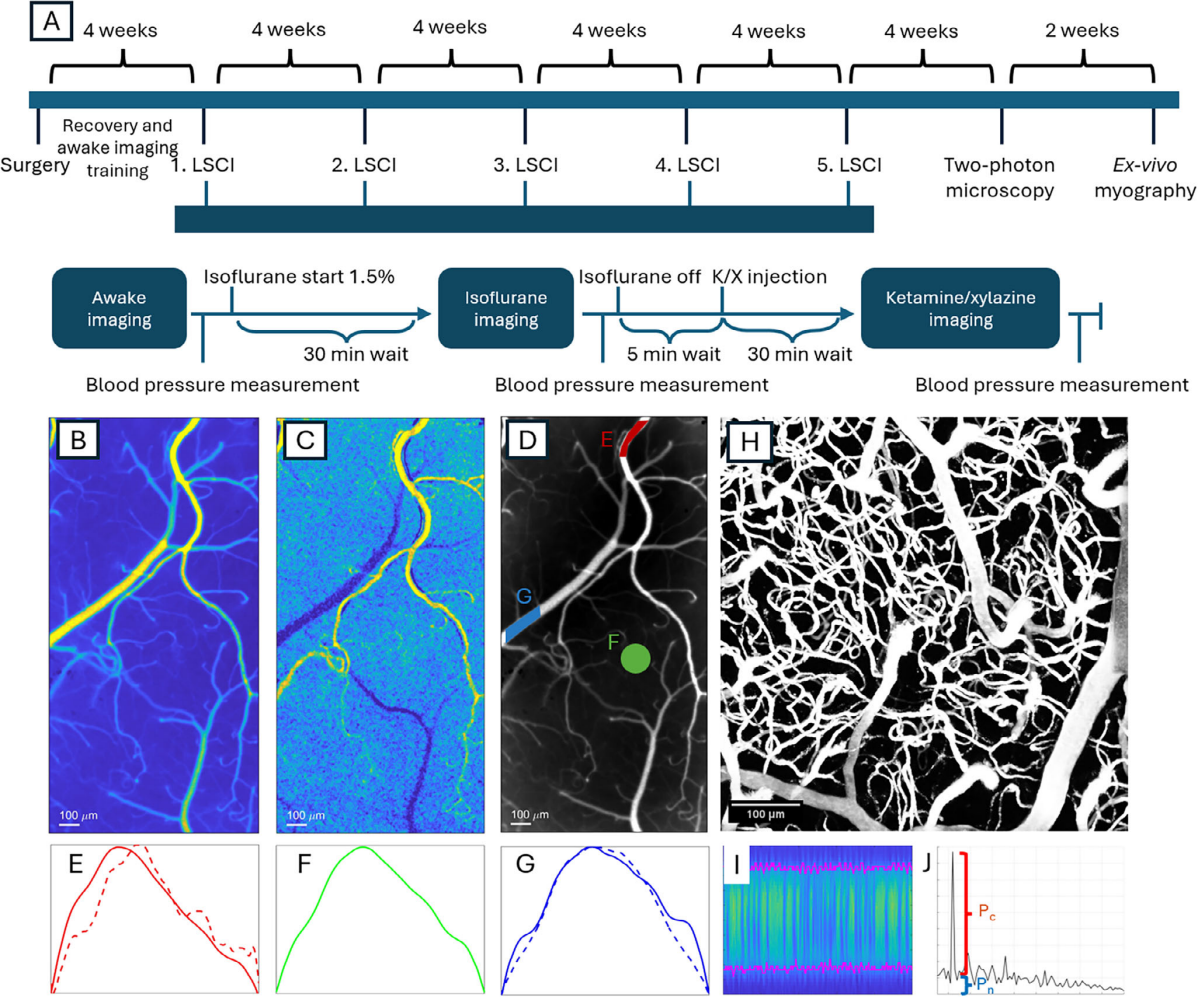
Experimental protocol and pulsatility imaging. A) ‐ timeline of procedures (top row) and the 3‐step LSCI recording protocol (bottom row) for each mouse. B) ‐ a representative average *BFI* image. C) ‐ a representative *PI*_*BFI*_ image of the same mouse as in B. D) ‐ example of arterial (red), parenchyma (green), and venous (blue) ROIs placement. E–G) ‐ examples of the BFI (solid lines) and diameter (dashed lines) changes over the representative cardiac cycle in the corresponding ROI types in D. H) ‐ two‐photon microscopy angiogram. I) ‐ an example of a cross‐sectional capillary line scan is one in which the X‐axis represents time and the Y‐axis represents coordinates along the scan. The magenta color denotes the detected vessel boundaries used for diameter estimation and consecutive power spectrum analysis. J) ‐ an example of the Lomb–Scargle periodogram of capillary diameter dynamics estimated from the line scans. *P*_*c*_ and *P*_*n*_ reflect the peak prominence and the noise pedestal at the cardiac frequency.

Each imaging session consisted of awake, isoflurane anesthesia, and ketamine/xylazine anesthesia for a total experiment time of up to ≈3 h (**Figure** **8**A). Before imaging, the mice were lightly anesthetized for 3 min in 3% isoflurane in 0.8L min^−1^ oxygen and moved to the imaging stage with a homeothermic heating pad to maintain a core body temperature of 37°C during the anesthetized sessions. The mice were head‐fixed to the imaging stage using clamps attached to the metal head plate. Once restrained in the imaging setup, a tail cuff (Kent Scientific) was placed on the mouse's tail to allow blood pressure measurement. The cuff was then gently taped to the heating pad to keep it in place and warm the tail. The mice were allowed to recover from the initial isoflurane anesthesia induction for at least 15 min, but typically 20–30 min, while setting up the tail‐cuff and focusing before the experiment started.

In each LSCI session, mice were imaged under three conditions while keeping the exact same field of view (Figure 8A): awake, isoflurane (1.5%), and ketamine‐xylazine (75 mg kg^−1^ ketamine, 10 mg kg^−1^ xylazine) with 30 min between each condition. Respective blood pressure measurements were taken at the end of each recording. Following the awake recording, a gas mask was placed over the mouse's head, and the mouse was induced with 3% isoflurane and 0.8 L min^−1^ oxygen for 3 min. Isoflurane was then decreased to 1.5%, which was found to be the lowest concentration at which stable blood flow can be achieved in our experimental setup. At lower values, *BFI* strongly fluctuated, most likely reflecting instabilities in anesthesia depth, breathing rate, and, therefore, isoflurane concentration in the bloodstream. The LSCI recording was performed after blood flow had stabilized, but no less than 20 min after induction of anesthesia. Isoflurane ventilation was then stopped, and the mask was removed after the respective blood pressure measurement was completed. After the mouse showed signs of recovery from anesthesia (but not less than 15 min), the ketamine‐xylazine anesthesia cocktail was injected intraperitoneally. LSCI data were recorded 30 min after this injection. The procedure described above was repeated every four weeks for five months for each mouse. It is important to note that the 3‐step protocol described above was chosen to minimize the number of imaging sessions and stress for the mice. Recovery after the isoflurane anesthesia is typically within minutes,^[^ ^78^ ^]^ so it has always preceded ketamine‐xylazine, whose effect lasts much longer, making a randomized order experiment design unfeasible. Pilot trials were have also performed without isoflurane to account for its possible impact on ketamine‐xylazine anesthesia and found no significant differences in the brain perfusion parameters (data not shown). To specifically investigate sex differences, a modified protocol, consisting exclusively of LSCI recordings in the awake state, without subsequent anaesthesia, two‐photon microscopy, or ex vivo experiments, was used in a separate cohort of mice.

### Laser Speckle Contrast Imaging of Microvascular Pulsatility—Imaging system and data acquisition

A custom LSCI system was designed to ensure high repeatability, precision, and accuracy of longitudinal imaging experiments. Specifically, the key factors are aimed to improve that potentially increase measurement variation in the conventional LSCI: inhomogeneous illumination, low coherence degree, varying polarization, and speckle size.^[^ ^79^, ^80^ ^]^ To achieve it, a coaxial illumination LSCI system based on a polarizing beamsplitter cube was developed. A highly coherent, volume holographic grating stabilized laser diode (785 nm, Thorlabs FPV785P) coupled to a polarization‐maintaining fiber was used to achieve a stable and highly homogeneous light output with a fixed polarization orientation. The laser was driven by a combined laser diode and temperature controller (CLD1015, Thorlabs). The light was collimated (F810APC‐780, Thorlabs) and delivered onto a polarizing beam‐splitting cube (CCM1‐PBS252/M, Thorlabs) via a custom‐built adjustable 1:1 Galilean telescope and a stirring mirror. The laser polarization was adjusted so the beam‐splitting cube would reflect nearly 100% of the light, directing it to the infinity‐corrected objective (Leica 2.5 N Plan, NA = 0.07). The stirring mirror and the telescope were adjusted to achieve homogeneous illumination at the objective's working distance. The light scattered by the object would be collected and directed to the beam‐splitting cube, where cross‐polarized light is transmitted onto the infinity corrected tube lens (TTL200‐B) and then to the CMOS camera (Basler aca2040‐90um NIR, 5.5 × 5.5 µ*m* ^2^ pixels). A neutral‐density filter was inserted in the illumination arm to ensure the average light intensity on the sensor would be ≈35% of the saturation when recorded at 5000 µ*s* exposure time.^[^ ^79^ ^]^ After the initial adjustments, every system component was locked, ensuring no deviations throughout the study. Calibration checks were also performed by measuring the coherence degree with a static phantom several times during the project to ensure no changes occurred in the system. In each experiment, the LSCI recordings lasted for 600 s and were taken at a frame rate of 194 frames per second and an exposure time of 5000 µ*s*. Due to the data storage capacity, the field of view was limited to 1024 × 512 pixels and ≈2.5 × 1.25 mm, therefore producing ≈58.2*GB* data per recording and ≈10.5*TB* in total.

For the sex‐comparison part of the study, the imaging configuration was modified to expand the field of view to ≈3 × 1.8 mm by employing a lower magnification objective (Thorlabs TL2X‐SAP, 2x, NA = 0.1) and a 1496x896 pixel acquisition window. Accordingly, the recording duration was shortened to 300 s to ensure a manageable data volume. Note that this modification of the objective alters the speckle size, thereby influencing the absolute BFI values.

### Laser Speckle Contrast Imaging of Microvascular Pulsatility—Data analysis

LSCI pulsatility analysis comprises several steps, starting with the estimation of the representative cardiac cycle, followed by pulsatility‐index guided placement of vessel‐type‐specific regions of interest (ROIs), and completed by dynamic segmentation of the blood vessels within the ROIs. The representative cardiac cycle was estimated with the following steps:

1) The field of view is masked to exclude artifacts and areas outside of the cranial window
2) The average spatial contrast for each frame is calculated to generate a single refined time course of contrast across the entire field of view. Spatial contrast analysis was described in detail before.^[^ ^81^, ^82^, ^83^ ^]^ Briefly, the contrast for each pixel was calculated as $K=\frac{\sigma (I)}{<I>}$, where σ(*I*) and <*I* > are the standard deviation and mean of intensity in the 5 × 5 neighborhood surrounding the pixel. The contrast calculated for individual pixels, except those masked out in the first step, was then averaged across the frame to produce the average contrast time course. In the present study, each time course consisted of 116 400 points, corresponding to 194 frames per second for a duration of 600 s.
3) Individual cardiac cycles are identified from the average contrast time course. More specifically, the fast Fourier transform was first applied to identify the dominant frequency of the cardiac cycle, with which local minima corresponding was determined to the individual cycles while allowing some degree of heart rate variability.
4) Each cycle is characterized according to a set of features, including but not limited to average, standard deviation, magnitude, duration, the difference between contrast values at the beginning and the end, and the number of noise spikes (increases during the descent phase or decreases during the ascending phase).
5) Cycles that do not fit the data quality criteria for the abovementioned features are rejected. For most features, the rejection criterion was defined as a deviation from the median value by more than two standard deviations, providing a robust quality control measure.
6) Contrast data is interpolated in time such that every accepted cycle will contain the same number of frames defined as the median cycle duration multiplied by the chosen interpolation factor. It is used to avoid feature loss caused by variations in the cycle duration, effectively resulting in irregular sampling and allowing a “super‐resolution” temporal analysis approach. Notably, only a minor duration variation is permitted in non‐excluded cycles, which should not arise from significant changes in heart activity.
7) Average interpolated cycles to produce the representative contrast cycle *K*_*cycle*_ and, then, convert it to blood flow index as *BFI* = 1./(*K*_*cycle*_.^2^), a metric previously validated against other perfusion measurement techniques.^[^ ^82^, ^84^, ^85^, ^86^ ^]^

The exact computational implementation of the algorithm, along with all cycle rejection criteria, can be found on our GitHub.^[^ ^87^ ^]^ The implementation is designed to be adaptable, with additional steps included to reduce rapid access memory requirements, making it suitable for various research scenarios.

From the resulting BFI images, the per‐pixel pulsatility index was calculated as *PI* = (*BFI*_*max*_ − *BFI*_*min*_)/ < *BFI* >. Examples of average BFI image and corresponding PI image are shown in Figure 8B,C, and Figures S1 and S2 (Supporting Information). The PI images were used to guide semi‐automated segmentation, where the user selects regions of interest (ROIs) containing segments of arteries, veins, or parenchyma. Arterial and venous ROIs are then used as an input for the dynamic segmentation algorithm, modified from our previous studies,^[^ ^73^, ^75^ ^]^ which provides diameter and intra‐vessel *BFI* estimations at each frame. Briefly, the algorithm uses an average *BFI* image of the provided ROI to determine the approximate location of the vessel's centerline and its orientation. Based on it, the average *BFI* profile is calculated across the center line. Similar to the calculation of the representative cardiac cycle above, the center line is not perfectly aligned with pixels, effectively resulting in irregular sampling. Therefore, using interpolation during the average profile calculation allows a spatial “super‐resolution” approach and retention of the “sub‐pixel” information.^[^ ^73^ ^]^ The values in the average profile are then classified into vessel or background using the minimum intra‐class variation approach. Diameter and intra‐vessel *BFI* at every frame are then calculated accordingly (Figure 8E,G). Parenchymal ROIs are defined as 5000‐pixel‐sized regions between resolvable vessels and, therefore, do not require dynamic segmentation. Instead, the respective pixel values were directly used to calculate the ROI‐averaged BFI (Figure 8F).

Finally, following the described above pre‐processing, the respective ROI‐ and vessel‐averaged BFI values, as well as vessel diameter values were used to calculate corresponding BFI pulsatility index *PI*_*BFI*_ = (*BFI*_*max*_ − *BFI*_*min*_)/ < *BFI* > and diameter pulsatility index *PI*_*D*_ = (*d*_*max*_ − *d*_*min*_)/ < *d* >. These four parameters (<*BFI* >, <*D* >, *PI*_*BFI*_, and *PI*_*D*_) were used to provide an in‐depth characterization of microvascular pulsatility in the aging mouse cortex presented in the Results section.

### Two‐Photon Microscopy

Following the last LSCI timepoint, two‐photon microscopy (TPM) was used to assess capillary density, diameter, and pulsatility in 9 out of 12 mice (3 in each age group). All TPM recordings were performed in awake mice. A tail vein catheter for infusion of fluorophores was placed under anesthesia before the imaging session. Anesthesia was induced with 3% isoflurane in 0.8L/min oxygen and maintained at 1.5‐2% during catheter insertion. During anesthesia waning, mice were fixed to the imaging stage to prepare for awake‐restrained imaging. Imaging was performed on an Investigator‐IV two‐photon system (Bruker Corporation, Billerica, MA, United States) with PrairieView software version 5.5 (Bruker Corporation). A load of 300µ*L* of 0.5% (0.5mg mL^−1^) solution of Texas Red 70kDa was administered via a tail vein catheter for vessel visualization and z‐stack acquisition. The data were acquired using two scanning protocols: an entire field‐of‐view scan to obtain angiograms and cross‐sectional line scans over random capillary branches. For angiograms, a 25x objective (Olympus, NA = x, WD = 8mm) was used to acquire z‐stacks of ≈190µ*m* (FOV = 515x512, 461.8µ*m* ^2^, laser excitation 950nm, 300 pockels power, 587 GaAsP). For each line scan, 3 to 4 cross‐sectional intensity profiles of the same capillary were captured at a rate of 300,000 pixels per second. Line scans were recorded for 30 s, with a 1‐2 ms scan line period, depending on the exact number of pixels per line.

TPM‐acquired angiograms were reconstructed in ImageJ using bleaching correction, contrast enhancement with normalization (saturated pixels = 0.4%), background subtraction (rolling ball = 50), and 3D filtering (median 3D: x = 2, y = 2, z = 2) and presented as Z projected maximum intensity projections (MIP) and 3D renderings (3D viewer) as shown in Figure 8H. Capillary vessels density was then calculated using Deepvess: first, the contrast was enhanced in each image in the z‐stack and corrected for any motion present, then, the 3D skeleton of the vessels was extracted, and the volume of each vessel segment and the entire angiogram were calculated. Finally, the cross‐sectional line scans were used to estimate capillary diameters and pulsatility. Due to the specifics of the TPM data, capillary pulsatility analysis has been performed differently from LSCI and included the following steps:

1) Capillary boundaries, average intensity, and signal‐to‐noise ratio were roughly estimated over the entire scan.
2) The estimates obtained from the previous step were used to classify pixels as foreground (capillary) and background at every timepoint using k‐means segmentation. An example of the segmented vessel is shown in Figure 8I.
3) Coordinates of vessel boundaries were identified, and sections of the image likely containing artifacts were excluded. The diameter was then estimated for every non‐excluded section of the image.
4) The Lomb‐Scargle periodogram^[^ ^88^ ^]^ was used to calculate the power spectrum of fluctuations in diameter and boundary coordinates of the vessel. The periodogram is calculated using a 5‐s window for frequencies ranging from 1 to 30 Hz, and then averaged across the entire observation period and all cross‐sections belonging to the same capillary. Figure 8J shows an example of the resulting power spectrum. The Lomb‐Scargle periodogram was used because it allows us to calculate power spectra of signals with missing data, such as the coordinates and diameter estimates, following the exclusion of artifacts.
5) Pulsatility in diameter and vessel boundaries was characterized as signal‐to‐noise ratio (SNR) at the cardiac frequency. Specifically, $SNR=\frac{P_{c}}{P_{n}}$ (Figure 8J) was defined, where *P*_*c*_ is the prominence of the most prominent peak at the frequency range from 9 to 13Hz and *P*_*n*_ is the noise pedestal at the same frequency. A noise pedestal is defined as the difference between the peak's height and prominence; therefore, *SNR* > 1 means that the peak's power is at least twice that of the noise.

### Isometric Wire‐Myography and Histology

Mice were euthanized by cervical dislocation, and the brain was immediately dissected into ice‐cold salt solution (PSS in mM: 116 NaCl, 2.82 KCl, 1.18 KH2PO4, 25.0 NaHCO3, 0.03 EDTA, and 5.5 glucose; pH 7.4 adjusted at 37°C with NaOH and aerated with 5% CO2 in air). Middle cerebral arteries from the left hemisphere were gently isolated and mounted in an isometric wire myograph (Danish Myo Technology A/S, Denmark). Following a minimum of 30 min equilibration at 37°C in PSS aerated with 5% CO2 in the air, the cerebral arteries were normalized in LabChart (ADInstruments, Dunedin, New Zealand). The normalization was based on the construction of the passive wall tension to the internal circumference relation. The inner diameter, calculated from internal circumference values in relation to passive wall tension, was used to assess vascular wall compliance via the passive length‐tension relation in the vascular wall (Figure 2C).

At the end of the experiment, the arterial segments were fixed directly in the myograph chamber with ice‐cold 4% PFA, followed by 24 h of incubation, and then stored in sterile PBS at 4°C until paraffin‐embedded for histological analysis. The paraffin‐embedded middle cerebral artery segments were sliced for 5 µm and de‐paraffinized (twice 10‐min xylene exposure, twice 5‐min 99% ethanol exposure, once 5‐min exposure to 95% and then 70% ethanol, following distilled water). Slices were stained with Weigert's hematoxylin for 8 min, washed twice in water, and stained in Picro‐Sirius Red for 1 h. Then, slices were washed in 0.5 v/v% acetic acid, dehydrated in 99% ethanol, cleared in xylene, and mounted with Eukitt on a coverslip for imaging. Imaging was done using the Olympus VS120 slide scanner (Olympus, Tokyo, Japan) at 40× magnification. The analysis of images was performed using ImageJ (version 1.54f, NIH, USA). All image analyses were done blinded and with the same settings. An average red staining intensity in the vascular wall was calculated as a proxy for collagen content.

### Statistical Analysis

Statistical significance was tested with one‐way ANOVA in all of the imaging experiments. The extra sum‐of‐squares F test was used for the myography tension curves. The exact *p* − *values* were reported, except for *p* < 0.0001. Unless noted otherwise, data are shown as mean ± standard deviation, and the null hypothesis was rejected for values of p<0.05. Statistical analysis was carried out using MATLAB 2025a software.

## Conflict of Interest

The authors declare no conflict of interest.

## Supporting information

Supporting Information

## Data Availability

The data that support the findings of this study are available from the corresponding author upon reasonable request.
