# Supplementary material for: Cortical Microvascular Pulsatility in the Aging Mouse Brain and the Confounding Effects of Anesthesia
Source: Adv Sci (Weinh). 2025 Nov 7;13(4):e19324. doi: 10.1002/advs.202519324 (PMC12822393; doi:10.1002/advs.202519324)
Supplement: Supplementary file 1 — Supporting Information [file ADVS-13-e19324-s001.pdf]

Supporting information. Cortical Microvascular Pulsatility In The Aging Mouse Brain And The Confounding Effects Of Anesthesia

*Mia Viuf Skøtt Elizaveta Melnikova Eugenio Gutiérrez Vladimir Matchkov Leif Østergaard Dmitry D Postnov**

Mia Viuf Skøtt, Leif Østergaard, Dmitry D Postnov*
Center of Functionally Integrative Neuroscience, Department of Clinical Medicine, Aarhus University, Aarhus, Denmark
dpostnov@cfin.au.dk
Elizaveta Melnikova, Eugenio Gutiérrez, Vladimir Matchkov
Department of Biomedicine, Aarhus University, Aarhus, Denmark

Supplementary Figure 1 - average BFI images.

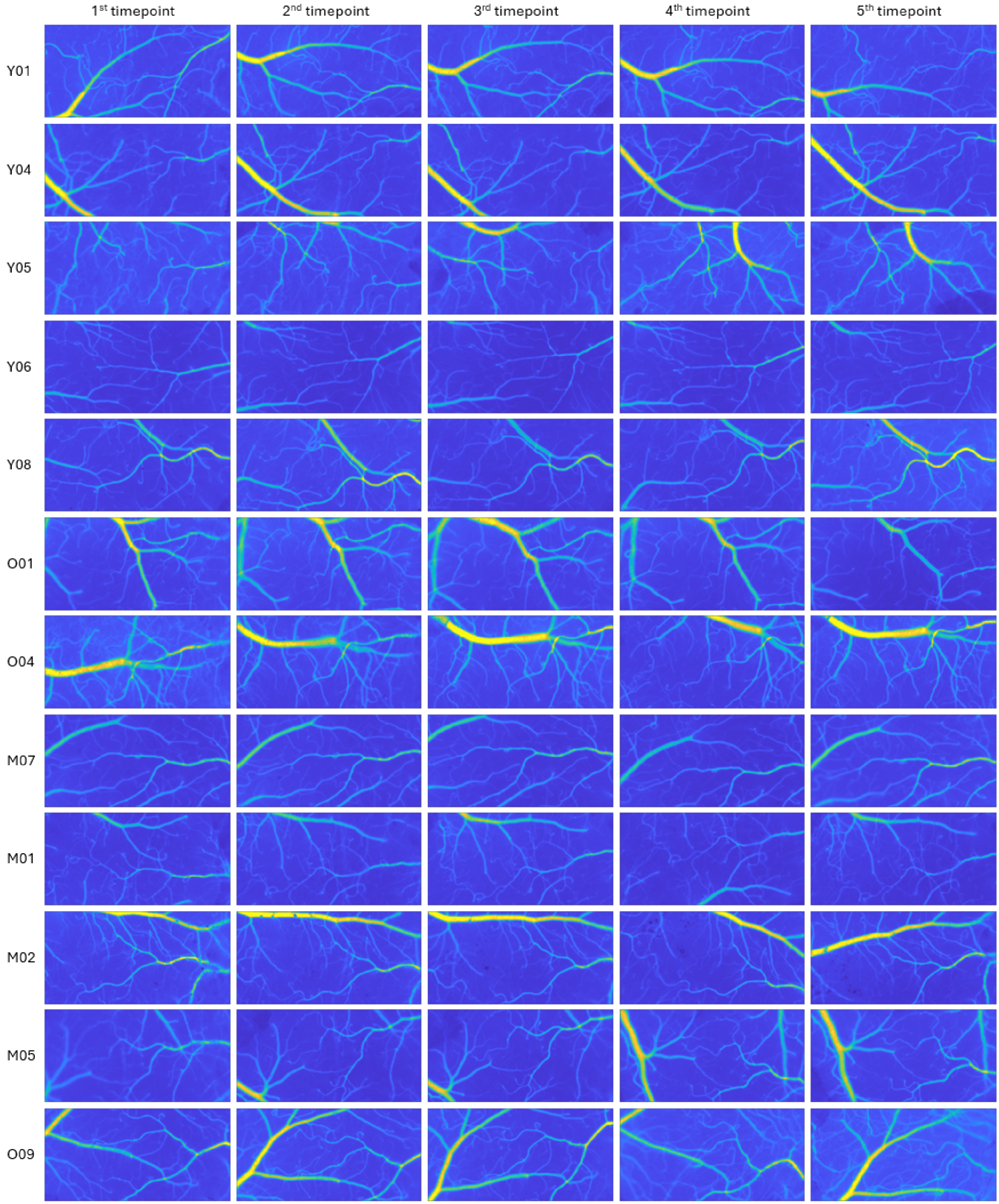

Figure 1: Average blood flow index images for all awake recordings - columns correspond to different timepoints, rows to different mice. The colormap limits are identical in all images.

Supplementary Figure 2 - average PI images.

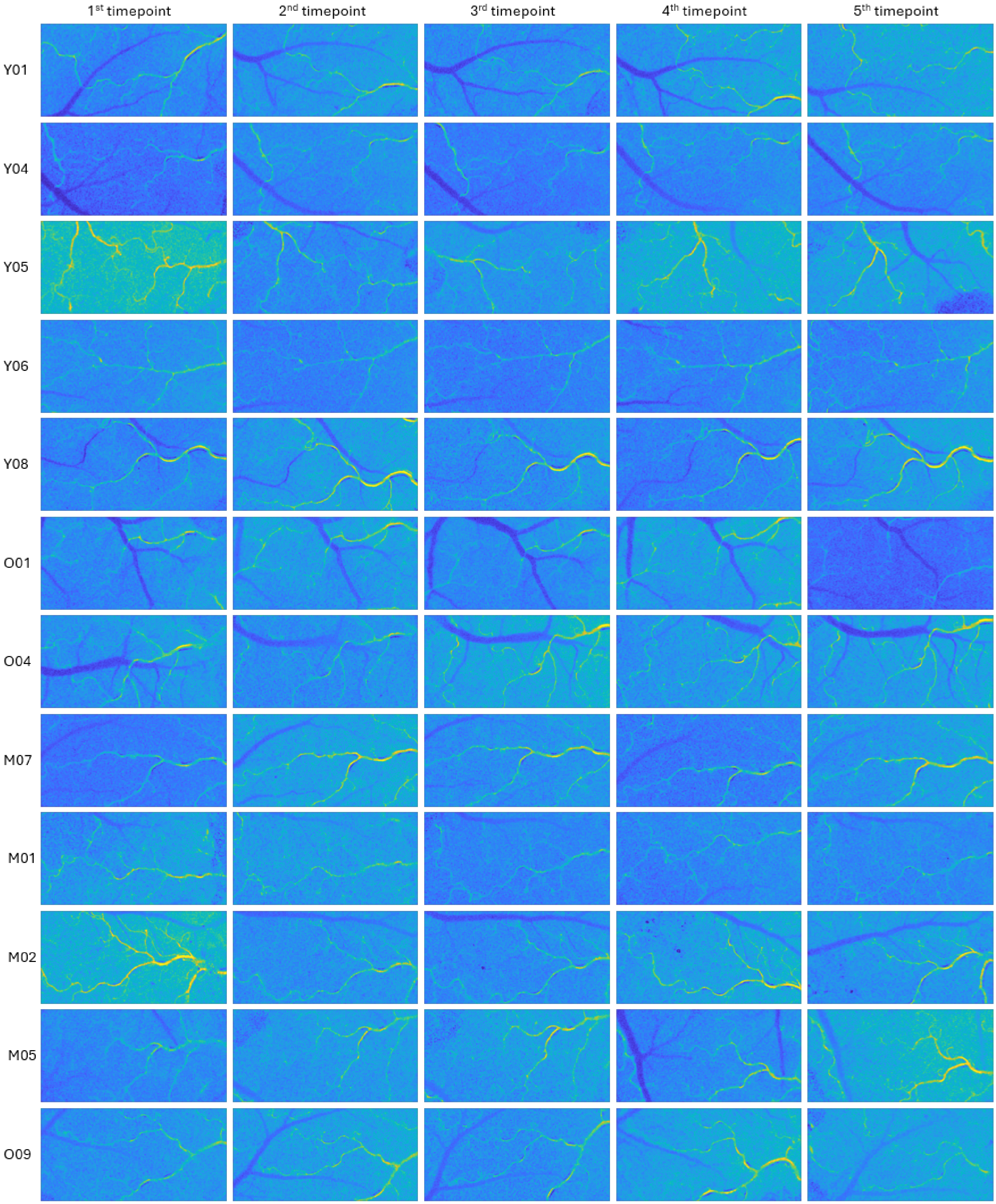

Figure 2: Average pulsatility index images for all awake recordings - columns correspond to different timepoints, rows to different mice. The colormap limits are identical in all images.

Supplementary Figure 3 - number of mice and regions of interest for each timepoint.

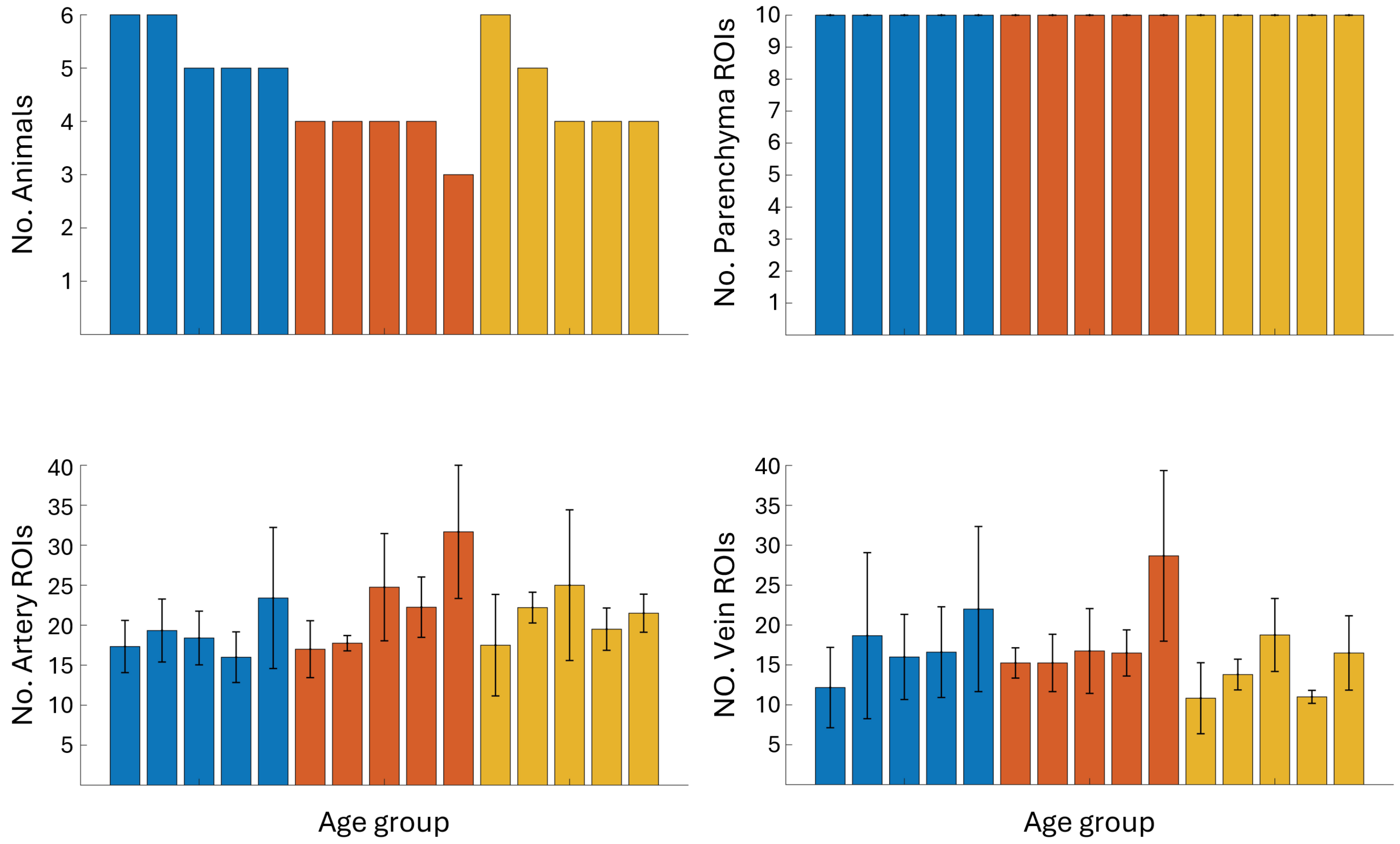

Figure 3: Number of mice and respective regions of interest for each timepoint. A – number of mice. Note that the same mice are used for different timepoints within the age group. B – number of parenchymal regions. C – number of segmented arteries and arterioles. D – number of segmented veins and venules.

Supplementary Figure 4 - measurements correlation in awake mice.

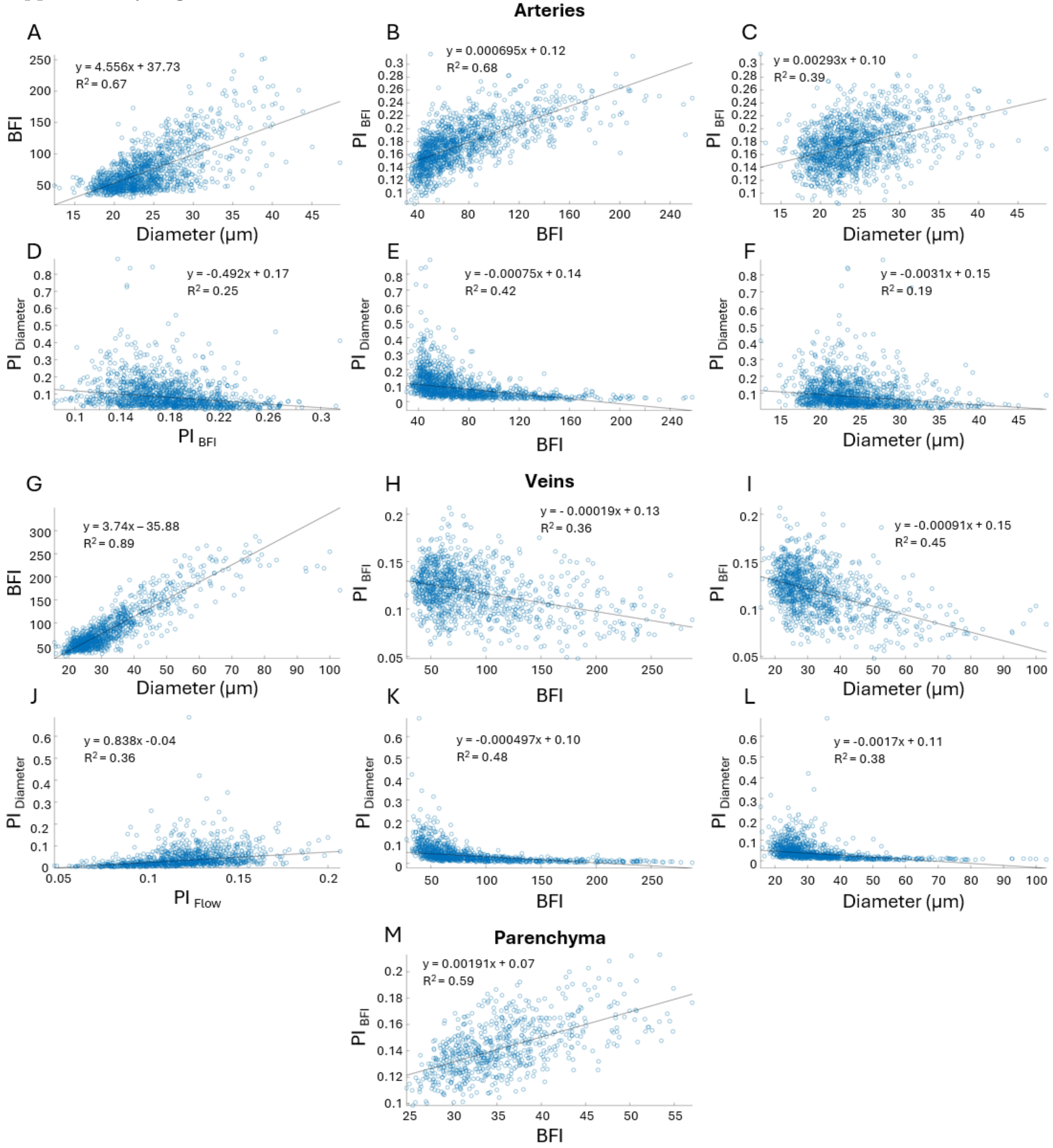

Figure 4: Measurements correlation in awake mice for different regions of interest. Parameters (a and b) of respective linear regression models ($y = a + bx$) and the goodness of fit metric (R^2) are stated in the legend for each graph.

Supplementary Figure 5 - TPM and LSCI and dynamics examples.

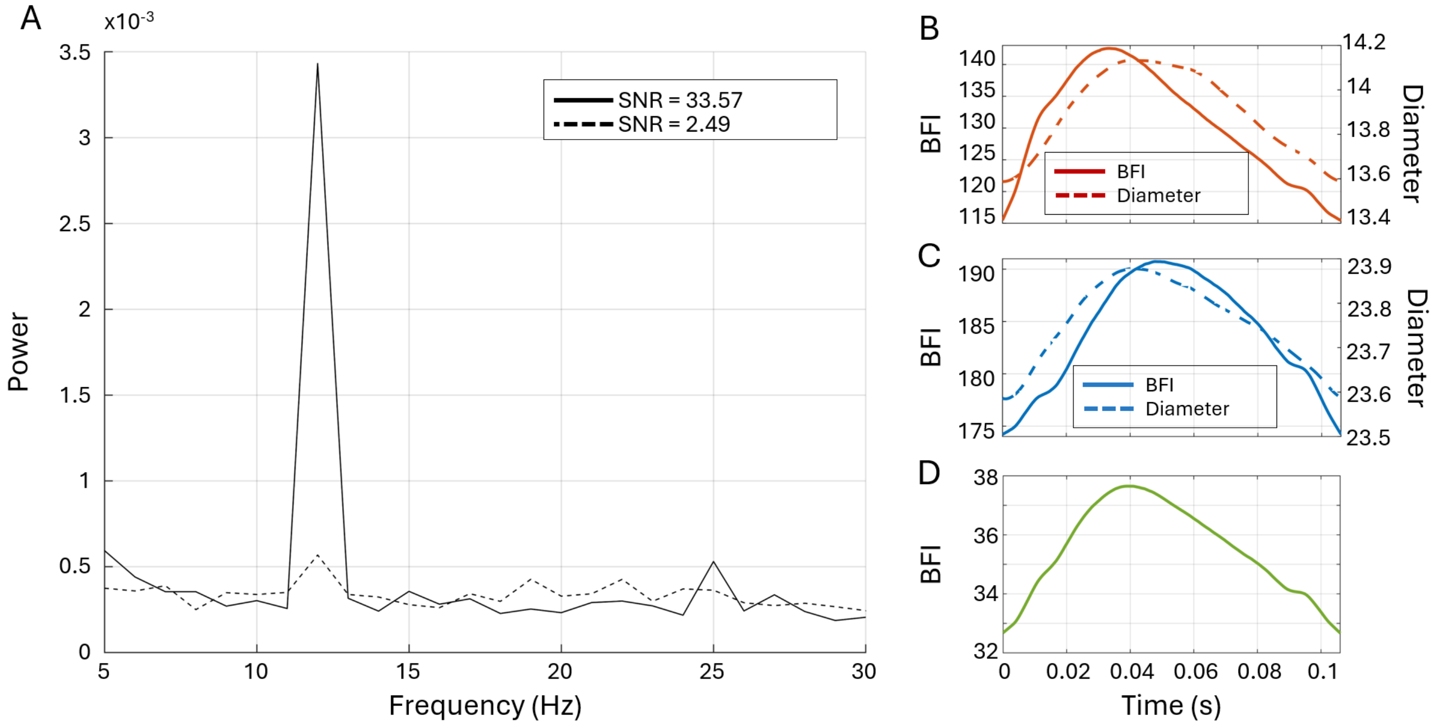

Figure 5: A - examples of Lomb-Scargle periodograms calculated from TPM line scans of strongly and weakly pulsating capillaries, respectively. B-D Examples of arterial, venous, and parenchymal BFI and diameter dynamics during the cardiac cycle.

Supplementary Figure 6 - Change in pulsatility and perfusion across the microvascular network under anesthesia

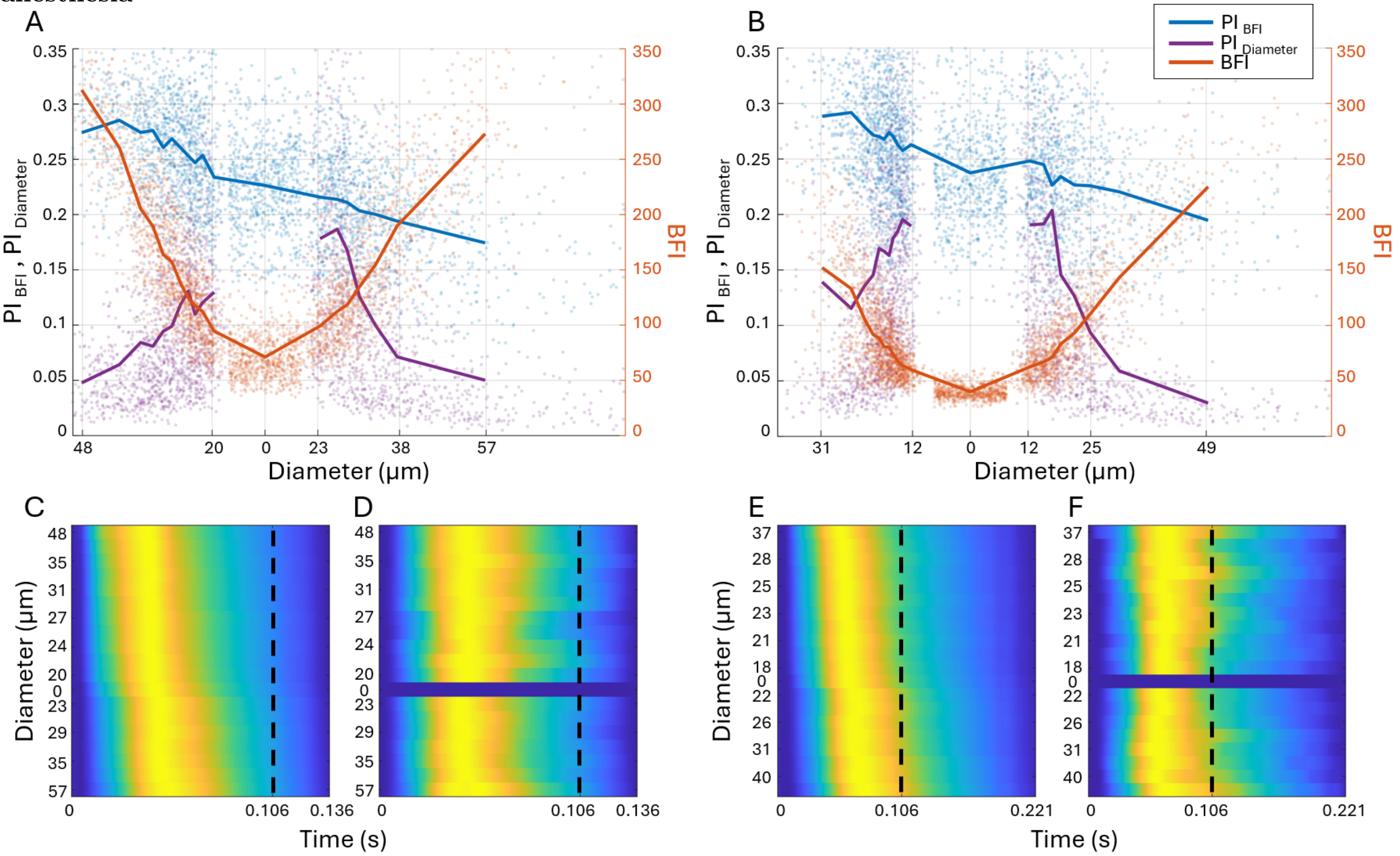

Figure 6: Average diameter and perfusion pulsatilities PI_D and PI_{BFI} (yellow and blue lines, left Y axis), and blood flow index BFI (red, right Y-axis) plotted as a function of the average diameter D across the imaged microvascular network. A – during isoflurane anesthesia. B – during ketamine-xylazine anesthesia. C, D - respective shapes of the BFI and diameter change during the cardiac cycle under the isoflurane anesthesia, normalized for each average diameter and presented as a color-coded map with time and diameter axis. E, F - respective shapes of the BFI and diameter change during the cardiac cycle under the ketamine-xylazine anesthesia, normalized for each average diameter and presented as a color-coded map with time and diameter axis. Note how values and shapes have changed compared to the measurements in the same regions of interest in awake mice (Fig. **??**). Most interestingly, the decline of the perfusion pulsatility has slowed down for isoflurane and ketamine-xylazine δPI_{BFI} between the largest arteries and veins is $\approx$ 0.1, while for awake mice, it was ≈ 0.15.
